# An antibody-drug conjugate designed through clone and isotype selection restricts the growth of CSPG4-expressing triple-negative breast cancer

**DOI:** 10.1038/s41698-026-01341-0

**Published:** 2026-03-07

**Authors:** Benjamina Esapa, Yi Liu, Alicia M. Chenoweth, Katie Stoker, Natalia Łabędź, Pablo Romero-Clavijo, Kristina M. Ilieva, Jennifer Trendell, Blanca Navarro-Llinas, Erin Suriawinata, Tobias Butcher, Ning Wang, Melanie Grandits, Lais C. G. F. Palhares, Alexandra McCraw, Silvia Crescioli, Annelie Johansson, Sheeba Irshad, Anita Grigoriadis, Patrycja Gazinska, Sophia Tsoka, Vijay Chudasama, James R. Baker, Andrew N. J. Tutt, Anthony Cheung, David E. Thurston, Sophia N. Karagiannis

**Affiliations:** 1 St. John’s Institute of Dermatology, School of Basic and Medical Biosciences, & KHP Centre for Translational Medicine, King’s College London, Tower Wing, 9th Floor, Guy’s Hospital, London, UK; 2 Breast Cancer Now Research Unit, School of Cancer and Pharmaceutical Sciences, King’s College London, Innovation Hub, Guy’s Cancer Centre, London, UK; 3 Department of Informatics, Faculty of Natural, Mathematical and Engineering Sciences, King’s College London, Bush House, London, UK; 4 Biobank Research Group and Section of the Biobank Medical Facility, Population Diagnostics Center, Łukasiewicz Research Network—PORT Polish Center for Technology Development, Wrocław, Poland; 5 Department of Chemistry, University College London, London, UK; 6 Cancer Bioinformatics, School of Cancer & Pharmaceutical Sciences, King’s College London, Guy’s Cancer Centre, London, UK; 7 Department of Oncology and Haematology, Faculty of Medicine, Wroclaw University of Science and Technology, Wrocław, Poland; 8 The Breast Cancer Now Toby Robins Research Centre, The Institute of Cancer Research, London, UK; 9 Institute of Pharmaceutical Science, School of Cancer and Pharmaceutical Sciences, King’s College London, London, UK

**Keywords:** Cancer, Drug discovery, Oncology

## Abstract

Antibody–drug conjugates (ADCs) demonstrate therapeutic potential, but aggressive triple-negative breast cancers (TNBCs) require precise target selection and antibody optimisation. We identified chondroitin sulfate proteoglycan 4 (CSPG4) expression in neoadjuvant treatment-resistant TNBC to guide ADC development. Three anti-CSPG4 IgG1 antibodies with distinct variable regions (225.28S, 763.74, and 9.2.27) were engineered and compared. 225.28S IgG1 demonstrated the most efficient internalisation and potent cancer cell cytotoxicity when conjugated to the tubulin inhibitor MMAE. To determine the optimal isotype, we generated 225.28S IgG4 and directly compared it with 225.28S IgG1. The IgG1 isotype showed superior internalisation and killing activity as an MMAE-conjugated ADC. Conjugation of 225.28S IgG1 to the topoisomerase inhibitor DXd produced an ADC with a drug-to-antibody ratio (DAR) of 8. This ADC was capable of robust internalisation into cancer cells and tumour cell cytotoxicity in vitro, and significant growth restriction of two CSPG4-expressing TNBC patient-derived xenografts (PDX) implanted orthotopically in mouse mammary fat pads. Unconjugated 225.28S IgG1 also limited TNBC xenograft growth in immunodeficient mice engrafted with human immune cells, confirming Fc-mediated functional activity. These studies identify 225.28S IgG1 as the optimal clone and isotype, supporting a next-generation DXd-conjugated ADC as a promising therapeutic strategy for hard-to-treat CSPG4-expressing TNBC.

## Introduction

Triple-negative breast cancers (TNBCs), accounting for approximately 15% of breast cancers, lack oestrogen and progesterone receptors (ER and PR), and are devoid of human epidermal growth factor 2 (HER2) expression^1–3^. Higher rates of proliferation and metastasis are clinical features of TNBC, contributing to poorer prognoses compared to other breast cancer subtypes^4^. The identification and development of novel targeted therapies is required to address this clinical need^5,6^. Antibody-drug conjugates (ADCs) are an emerging class of therapies for a range of malignancies, including breast cancers^7^. Apart from HER2-targeted ADCs trastuzumab emtansine (Kadcyla^®^) and trastuzumab deruxtecan (Enhertu^®^), two ADCs based on anti-TROP2 antibodies conjugated to topoisomerase inhibitors have been approved for the treatment of breast cancer: sacituzumab govitecan (Trodelvy^®^) conjugated to SN-38, for TNBC; and datopotamab deruxtecan (Datroway^®^) conjugated to DXd, for hormone receptor-positive breast cancer^8^. These approvals highlight the potential for developing ADCs targeting breast cancer antigens beyond HER2^8^.

In addition to TROP2, several other surface antigens have been explored for ADC development in TNBC, reflecting an effort to expand the therapeutic armamentarium. For example, B7-H3 (CD276), an immune modulatory/checkpoint molecule, is the target of the duocarmycin-based ADC MGC018, which has demonstrated preliminary antitumour activity in an early-phase clinical trial (NCT03729596)^9^. Receptor tyrosine kinase-like orphan receptor 1 (ROR1), a developmentally regulated oncofoetal antigen with limited expression in normal adult tissues, is the target of the anthracycline-based ADC NBE-002, which has shown manageable safety and early signals of efficacy in a phase I/II clinical study in patients with advanced TNBC (NCT04441099)^10^. These completed clinical trials highlighted substantial translational and developmental challenges that continue to accompany the identification of effective ADC targets in TNBC. More recently, BL-B01D1, a first-in-class ADC comprising an EGFR-HER3 bispecific antibody conjugated to a topoisomerase I inhibitor payload^11^, has progressed into phase II in patients with unresectable locally advanced or recurrent metastatic TNBC (NCT06471205). This reflects ongoing attempts to improve target coverage and therapeutic efficacy. Besides, the clinical success of ADCs can be influenced by antigen stability and resistance mechanisms. For example, TROP2-targeted therapies may be compromised by acquired mutations such as T256R, which impairs membrane localisation and reduces binding of Trodelvy^®^^12^. These observations underscore the importance of identifying and validating additional antigens that could enable effective treatment for patients whose tumours exhibit low expression of established targets or develop resistance to existing ADCs.

The development of effective ADCs requires the identification of a target antigen expressed by malignant cells and the selection of target-expressing patient cohorts amenable to the treatment. Originally reported as a melanoma-associated antigen, the transmembrane protein chondroitin sulfate proteoglycan 4 (CSPG4) has been implicated in pro-tumour and metastatic signalling pathways in several cancer types^13,14^. We previously reported an anti-CSPG4 ADC^15^ conjugated to a DNA-interactive pyrridinobenzodiazepine (PDD) payload^16^, which exhibited antitumour effects against human CSPG4-expressing melanomas^17^.

Other studies have also highlighted CSPG4 as a potential target for TNBC therapy, where the expression was identified in patient pleural effusions^18^ and correlated with a shorter time to recurrence^19^. An anti-CSPG4 immunotoxin exhibited selective in vitro cytotoxicity in CSPG4-high TNBC cell lines^20^. Moreover, an anti-CSPG4 single-chain variable fragment (scFv) (clone 9.2.27) conjugated to the tubulin inhibitor monomethyl auristatin F (MMAF) demonstrated promising in vitro internalisation and cytotoxic effects^21^. However, limited evidence is available on the expression pattern and distribution of CSPG4 in TNBC, particularly in patients with treatment-resistant disease. This patient group represents a significant unmet need and is associated with a higher risk of recurrence and limited therapeutic options.

After identifying a suitable target, the development of effective ADCs also requires the selection of an appropriate antibody clone against the target antigen. The efficacy of internalising ADCs relies on effective uptake into the target cell and trafficking to the lysosomal compartment for release of the cytotoxic payload. Some studies have indicated that the internalisation efficiency of an antibody may be linked to its epitope. For example, anti-HER2 antibodies recognising epitopes that do not block HER2 heterodimerisation with other ErbB family members internalise more effectively than those that inhibit heterodimerization^22^. Similarly, the epitope of antibodies has been shown to impact immune cell-mediated killing of cancer cells. A study of Bispecific T-cell Engagers (BiTEs) targeting CSPG4 epitopes showed that those with plasma membrane proximity induced enhanced immune cell-mediated killing^23^. These findings underscore the importance of investigating different antibody clones against the chosen target antigen to develop highly effective ADCs.

The antibody isotype is another factor to consider in antibody-based therapeutics. All approved ADCs utilise antibodies of the IgG1 and IgG4 subclasses, with the majority being IgG1. IgG1 and IgG4 differ in the amino acid sequences of their hinge regions, with IgG4 possessing a shorter upper hinge region. Other key residues that differ in IgG4 (F234, S331, and G327) in the lower hinge and CH2 regions contribute to its reduced binding to FcγRs and the C1q receptor^24,25^. Yet, the impact of these structural differences on ADC internalisation remains unclear.

Therefore, in this study, we characterise CSPG4 expression in patients with primary and residual TNBC following neoadjuvant chemotherapy and identify an anti-CSPG4 antibody clone and isotype combination suitable for the development of antibody and ADC therapies.

## Results

### CSPG4 expression in TNBC primary tumour samples and following neoadjuvant chemotherapy

**Fig. 1:**
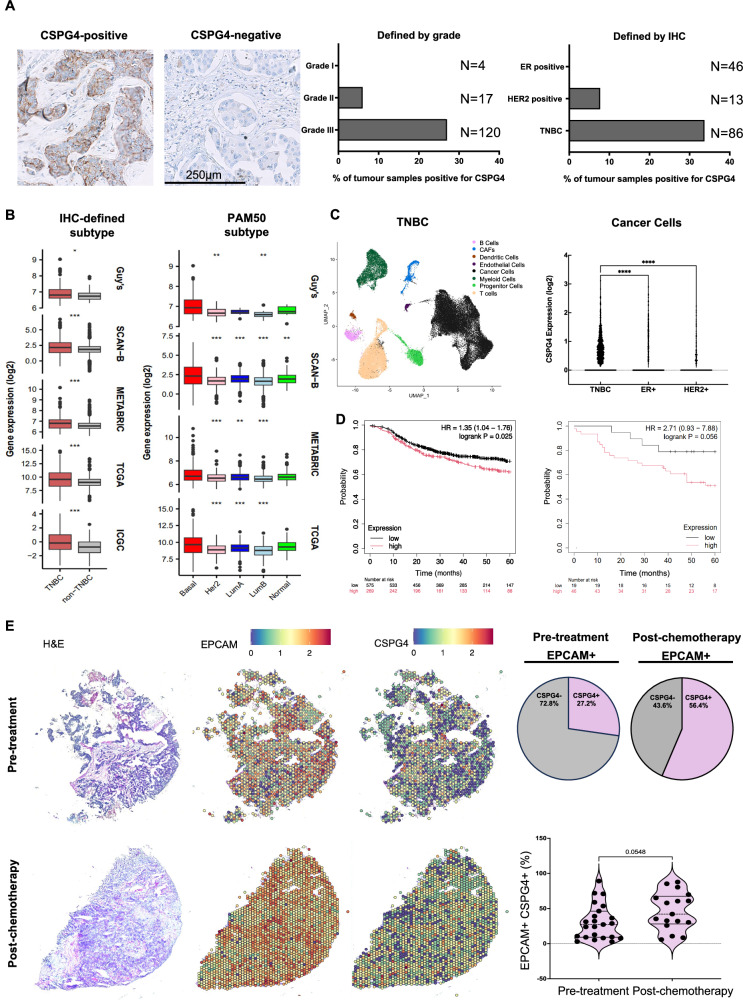
CSPG4 expression in breast cancer tissues. **A** CSPG4 expression by immunohistochemistry. Representative images (left), histograms of CSPG4 expression by grade and hormone-receptor status (right). **B** Bulk RNA-seq analysis of CSPG4 expression in breast cancers. Samples were stratified according to TNBC and non-TNBC status (left) and by the PAM50 criteria (right) according to hormone receptor status. Curated data were obtained from five databases (*n* = 6173 primary tumour samples (TNBC vs. non-TNBC)): Guy’s (*n* = 131 vs. 46), SCAN-B (*n* = 165 vs. 2942), METABRIC (*n* = 101 vs. 1096), TCGA (*n* = 112 vs. 824), ICGC (*n* = 73 vs. 186). CSPG4 expression stratified by PAM50 classification (Basal-like (Basal), HER2, luminal A (LumA), luminal B (LumB) and normal-like (Normal): Guy’s (*n* = 95, 28, 11, 10, 7), SCAN-B (*n* = 339, 327, 1657, 729, 221), METABRIC (*n* = 237, 181, 483, 383, 93) and TCGA (*n* = 232, 153, 345, 263, 91). PAM50 stratification was not available for the ICGC cohort, and statistical comparisons were made against TNBC or Basal-like breast cancer using the Mann–Whitney *U* test. **C** CSPG4 expression in a scRNA-seq dataset (GSE161529) of treatment-naïve breast cancer samples (cancer cells: EpCAM+). Histogram shows log-normalised CSPG4 expression level in each cancer cell cluster in TNBC (*n* = 8, 58,419 cells), ER-positive (*n* = 13, 63,554 cells) and HER2-positive (*n* = 6, 31,917 cells) samples. Expression levels were compared using the Kruskal–Wallis test with Dunn’s multiple comparisons. **D** Impact of low or high CSPG4 expression on relapse-free survival (RFS) on chemotherapy-treated breast cancer patients (left panel). Impact of low or high CSPG4 expression at the protein level across all breast cancer types (right panel). Kaplan–Meier plots generated using Kaplan-Meier plotter. **E** Spatial transcriptomic analysis of CSPG4 expression in TNBC pre- and post-chemotherapy. Investigation of the proportion of EpCAM-positive tumour areas also positive for CSPG4 expression in samples from treatment-naïve patients with TNBC (*n* = 23) and residual tumour (*n* = 17) samples following neoadjuvant chemotherapy. Left: Representative tumour slice images showing EpCAM and CSPG4 expression. Top right: Pie charts depicting overall proportion of CSPG4+EpCAM+ areas as % of all EpCAM+ areas across all samples in untreated and post-NAC samples. Bottom right: Comparison of % CSPG4+EpCAM+ areas per sample in treatment-naïve and post-NAC tumour groups. Statistical analyses were conducted using a two-tailed, unpaired, Mann–Whitney *U* test.

CSPG4 expression was investigated through immunohistochemical analysis of 142 primary breast cancer samples from treatment-naïve patients. Expression was associated with higher-grade disease, and TNBC samples showed the highest proportion of CSPG4 expression (~34%) (Fig. 1A).

Bulk RNA-seq analysis was also carried out across several patient cohorts, including the Breast Cancer Now Unit at King’s College London (*n* = 131), SCAN-B (*n* = 165), METABRIC (*n* = 101), TCGA (*n* = 112), and ICGC (n = 329). These confirmed that CSPG4 is significantly more highly expressed in TNBC compared to non-TNBC counterparts (Fig. 1B, Left Panel). Stratification by PAM50-defined subtypes^26^ confirmed elevated CSPG4 expression in basal-like/TNBC (Fig. 1B, Right Panel).

Single-cell RNA-seq data from treatment-naïve breast cancer patients^27^ (TNBC: *n* = 8 samples, 58,419 cells; ER-positive, *n* = 13 samples, 63,554 cells; HER2-positive, *n* = 6 samples, 31,917 cells) were interrogated. Cells were clustered using dimensionality reduction (tSNE), and cancer cell clusters were identified by positivity for Epithelial Cell Adhesion Molecule (EpCAM). In this dataset, TNBC cells showed a higher expression of CSPG4 than ER-positive or HER2-positive cancer cells (Fig. 1C). Notably, TNBC patient samples positive for CSPG4 expression were likely to co-express genes implicated in focal adhesion kinase (FAK) and mitogen-activated protein kinase (MAPK) pathways, known effectors of CSPG4 downstream signalling and implicated in tumour growth and metastasis (Supplementary Fig. 2A). Moreover, high CSPG4 expression was associated with poorer prognosis. At the protein level in treatment-naïve breast cancer patients, CSPG4 expression was linked with reduced survival (HR = 1.35, *p* = 0.025) (Fig. 1D, Left Panel). Similarly, transcriptomic data from chemotherapy-treated breast cancer patients showed a trend towards worse survival in CSPG4-high tumours (HR = 2.71, *p* = 0.056), supporting its association with pro-tumorigenic functions (Fig. 1D, Right Panel).

Given the strong association between CSPG4 and basal-like/TNBC subtypes, spatial transcriptomics profiling explored CSPG4 expression in TNBC before and after neoadjuvant chemotherapy (NAC). This included treatment-naïve TNBC samples (*n* = 23), and residual tumours post-NAC (*n* = 17)^28^. The proportion of EpCAM-positive tumour areas also positive for CSPG4 expression was investigated by studying “mini-bulk” RNA-seq areas of 5–10 cells within an area of 55 µm^2^ in tumour slices from untreated TNBC patients, compared to tumour slices from NAC-treated patients. Across all untreated TNBC samples, 27.2% of tumour areas were positive for CSPG4 expression, whereas for the NAC-treated TNBC patients, CSPG4 expression was measured at 56.4% of tumour areas (Fig. 1E). The mean percentage of CSPG4+EpCAM+ areas within an individual sample was found to be 30.5% in the pre-treated group and 46.5% in the NAC-treated group (Fig. 1E). Importantly, CSPG4-associated kinases were also retained in NAC-treated TNBC patient samples (Supplementary Fig. 2B).

Overall, these findings indicate that CSPG4 expression is retained or even enriched in residual TNBC following NAC, raising the possibility that chemotherapy-resistant TNBC could potentially be targeted with anti-CSPG4 therapies.

### Cellular binding and internalisation properties of anti-CSPG4 IgG1 clones

**Fig. 2:**
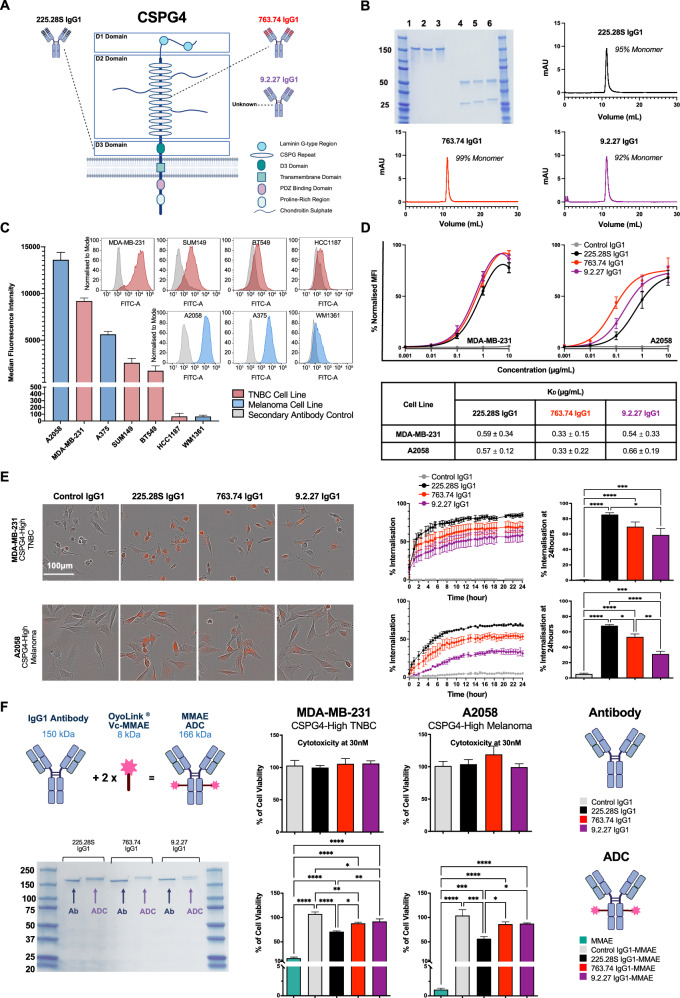
Evaluation of anti-CSPG4 antibody clones and ADCs. **A** Putative areas of epitope binding of anti-CSPG4 clones. Variable regions 225.28S are postulated to bind to the D3 domain of CSPG4, variable regions 763.74 bind to the D2 domain, and the precise binding site of variable regions 9.2.27 is not reported in the literature. Created using Biorender^63^. **B** Production and purity of anti-CSPG4 IgG1 clones. SDS-PAGE indicates intact antibody at 150 kDa under non-reducing conditions (Lanes 1–3), and the presence of bands consistent with the antibody heavy chain (50 kDa) and light chain (25 kDa) under reducing conditions (treatment with β-mercaptoethanol). Lane 1: 225.28S IgG1, Lane 2: 763.74 IgG1, Lane 3: 9.2.27 IgG1, Lane 4: Reduced 225.28S IgG1, Lane 5: Reduced 763.74 IgG1, Lane 6: Reduced 9.2.27 IgG1 (left). Size-exclusion chromatography traces of the three antibody clones are shown. **C** Identification of CSPG4-expressing TNBC and melanoma cell lines by flow cytometric evaluation using 225.28S IgG1 and FITC-conjugated anti-human IgG secondary antibody. Representative plots of fluorescence intensity compared to cells stained with secondary antibody only (non-specific binding), and quantitation of median fluorescence intensity for each cell line. **D** Flow cytometric assessment of isotype control (Control IgG1) and Anti-CSPG4 IgG1 clones binding to MDA-MB-231 and A2058 cells using FITC-conjugated secondary antibody. MFI values are normalised to the maximum MFI value for each experiment. *K*_D_ values and standard deviations are presented. **E** Investigation of internalisation of FabFluor®-labelled anti-CSPG4 IgG1 clones into MDA-MB-231 and A2058 cells at 24 h (one-way ANOVA with Tukey’s multiple comparisons test). Representative images and quantitation of fluorescence after 24-h incubation for cells treated with 30 nM of FabFluor-labelled antibody. **F** Generation of MMAE-conjugated ADCs and comparisons of cytotoxicity. Schematic representation of conjugation of IgG1 antibody (150 kDa) to 2 molecules of linker-payload (8 kDa), yielding an ADC product of 166 kDa. SDS-PAGE gel confirming higher molecular weight for anti-CSPG4 IgG1 clones after conjugation to payload. Left panel: Comparisons of cytotoxicity of MMAE payload, antibodies, and ADCs at 30 nM, with treatment over 96 h for MDA-MB-231 and A2058 cells (one-way ANOVA with Tukey’s multiple comparisons test, right panel). Created using Biorender^63^.

Following identification of CSPG4 expression in TNBC cells, we sought to identify anti-CSPG4 antibody variable regions with favourable properties for ADC development. Anti-CSPG4 variable regions from the murine antibody clones 225.28S^15^, 763.74^29^ and 9.2.27^30^ were cloned into a human IgG1 framework for comparative evaluations. The 225.28S clone variable regions have been postulated to bind to the D3 domain of CSPG4^31,32^, 763.74 variable regions have been shown to bind to a site in the D2 domain^33,34^, while the binding site of the 9.2.27 variable regions has not been reported (Fig. 2A).

Plasmids encoding the variable regions of each clone were generated in human IgG1 format using polymerase incomplete primer extension (PIPE) PCR as described previously^35,36^. Plasmid DNA was used to transfect Expi293F cells, and the resulting antibodies, 225.28S IgG1, 763.74 IgG1, and 9.2.27 IgG1, were purified from cell supernatants by affinity chromatography (Supplementary Fig. 1). SDS-PAGE confirmed the structural integrity of the antibodies, and SEC analyses indicated that all antibodies were produced at a high level of purity (>90%) and were free of significant aggregates (Fig. 2B).

For functional assessments, CSPG4-expressing cancer cell lines were evaluated by flow cytometry, leading to the selection of MDA-MB-231 (TNBC) and A2058 (melanoma) cells, which exhibited the highest cell surface expression of CSGP4 (Fig. 2C). All anti-CSPG4 IgG1 clones exhibited comparable binding affinity to both cell lines, with similar dissociation constants (*K*_D_), while the non-specific Control IgG1 did not bind to either cell line at any concentration (Fig. 2D).

The propensity of each antibody clone to internalise, a requirement for delivery of a cytotoxic payload, was assessed in the CSPG4-expressing target cells over 24 h. The three clones and the non-specific Control IgG1 were labelled with the pH-sensitive FabFluor^®^ dye, which only exhibits high levels of red fluorescence when it reaches an acidic cellular compartment such as a late lysosome. Quantification of the percentage of cancer cells showing a high level of red fluorescence was measured to observe internalisation and lysosomal localisation. The results revealed that 225.28S IgG1 exhibited superior internalisation kinetics over 24 h compared with 763.74 IgG1 and 9.2.27 IgG1, but minimal internalisation for non-targeting Control IgG1 (Fig. 2E).

### Cytotoxicity of MMAE-conjugated anti-CSPG4 IgG1 clones

To investigate whether the superior internalisation of 225.28S IgG1 translated to enhanced intracellular delivery of a cytotoxic payload and subsequent cytotoxicity against CSPG4-expressing cancer cells, a commercial conjugation kit (OyoLink^®^ Vc-MMAE, AlphaThera) was used to conjugate the antibodies. The OYoLink^®^ kit comprises the MMAE payload attached to a valine-citrulline linker, which is cleavable by lysosomal proteases. The linker-payload is joined to a UV-sensitive adaptor protein, which, after incubation with an antibody under 365 nm UV light, binds to a single site on each IgG heavy chain to confer a theoretical DAR of 2^37^. SDS-PAGE was used to provide an indication of whether the linker-payload had successfully conjugated to the antibody. As each linker-payload molecule has a molecular weight of approximately 8 kDa, the addition of two molecules per antibody results in a sufficient increase in molecular weight (i.e., 16 kDa) to be apparent from band positions on the gel. As expected, each ADC prepared from the three clones and the control antibody showed an increase in molecular weight (i.e., higher bands on the gel) compared to the unconjugated antibodies (Fig. 2F).

Next, the cytotoxic properties of the MMAE-conjugated ADCs were investigated over a 96-h incubation period in the CSPG4 + MDA-MB-231 and A2058 cell lines. Both MDA-MB-231 and A2058 cell lines exhibited sensitivity to MMAE, as treatment with free MMAE at 30 nM reduced cell viability to 16.79% and 1.06%, respectively (Fig. 2F). While the unconjugated antibodies exhibited no cytotoxic effects under treatment at 30 nM, treatment with 225.28S IgG1-MMAE at 30 nM resulted in 30% and 60% reductions in the viabilities of the MDA-MB-231 and A2058 cells, respectively, compared to PBS controls. Treatment with the 763.74 IgG1-MMAE and 9.2.27 IgG1-MMAE ADCs in equivalent conditions resulted in losses of viability of approximately 10% in MDA-MB-231 cells and 13% in A2058, while Control IgG1-MMAE did not affect cell growth in either cell line (Fig. 2F). Overall, these results suggested that 225.28S IgG1 may be superior to the other clones in delivering the MMAE cytotoxic payload, likely due to the superior internalisation rate (Fig. 2E).

### An antibody with the 225.28S variable regions exhibits superior internalisation and delivery of payload to cancer cells in an IgG1 format compared to IgG4

**Fig. 3:**
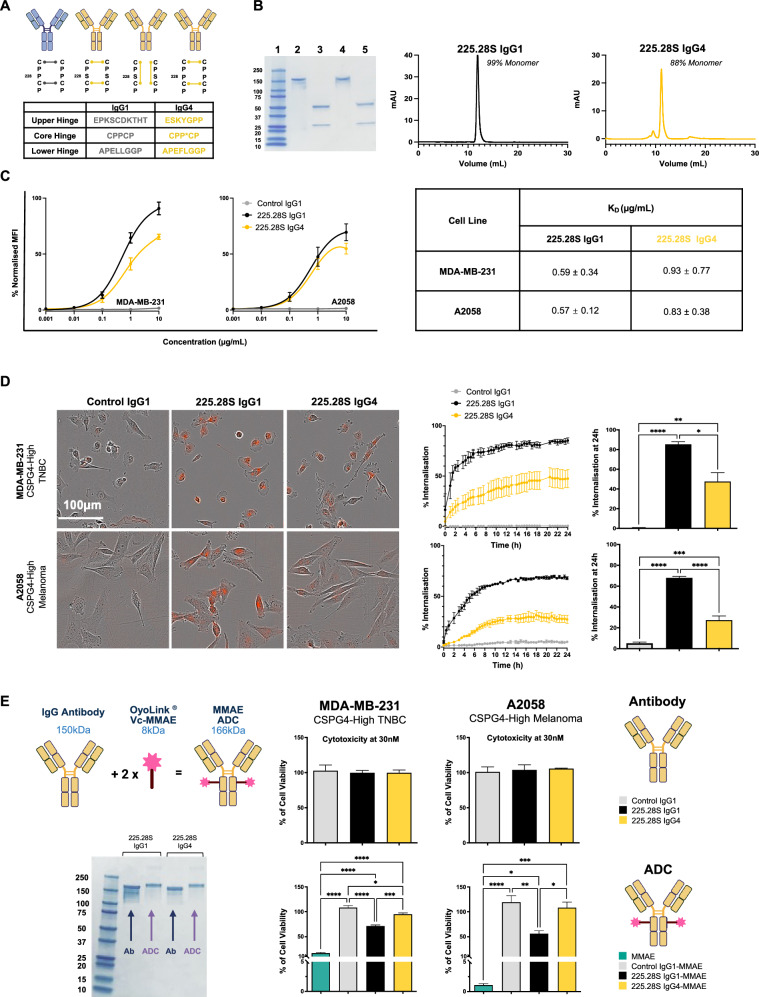
Evaluation of 225.28S IgG1 and IgG4 antibodies. **A** Schematic of structural differences in the IgG1 and IgG4 hinge regions, depicting an IgG1 antibody and the interchain disulfide bonds formed between cysteine residues in the core hinge (first antibody from the left, in blue). IgG4 antibody showing interchain disulfide bonds forming between heavy chain cysteine residues (second antibody from the left, in yellow). IgG4 showing intrachain disulfide bonds forming between cysteine residues within the same heavy chain, leaving the heavy chains held together only by non-covalent interactions (third antibody from the left, in yellow). IgG4 antibody containing the S228P mutation in which inter-chain but not intra-chain disulfide bonds form between the cysteine residues at the core hinge region (antibody on the far-right, in yellow). Table depicting differences in amino-acid composition of the hinge regions of IgG1 and IgG4. Created using Biorender^63^. **B** Production and purity analysis of Anti-CSPG4 IgG1 and IgG4 antibodies. SDS-PAGE gel Lane 1: Marker, Lane 2: Anti-CSPG4 IgG1, Lane 3: Reduced Anti-CSPG4 IgG1, Lane 4: Anti-CSPG4 IgG4, Lane 5: Reduced Anti-CSPG4 IgG4 indicated intact antibodies at 150 kDa and heavy and light chain in reducing conditions at 50 kDa and 25 kDa, respectively (left). Size Exclusion chromatography traces of the antibodies are shown (right). **C** Flow cytometric assessment of binding of isotype control (Control IgG1), Anti-CSPG4 IgG1, and Anti-CSPG4 IgG4 to MDA-MB-231 and A2058 cells using FITC-conjugated secondary antibody. MFI values are normalised to the maximum MFI value for each experiment. K_D_ values and standard deviations are shown. **D** Investigation of internalisation of FabFluor^®^-labelled Anti-CSPG4 IgG isotypes into MDA-MB-231 and A2058 cells. Representative images and quantitation of fluorescence after 24 h incubation of cells treated with 30 nM of labelled antibody. Internalisation at 24 h was compared using one-way ANOVA with Tukey’s multiple comparisons test. **E** Generation of MMAE-conjugated ADCs and comparisons of cytotoxicity. Schematic representation of conjugation of IgG1 antibody (150 kDa) to 2 molecules of linker-payload (8 kDa), yielding an ADC product of 166 kDa. SDS-PAGE confirming the increase in molecular weight for the anti-CSPG4 IgG1 and IgG4 after conjugation to the payload. Comparisons of cytotoxicity of antibodies and ADCs at 30 nM, with treatment over 96 h in MDA-MB-231 and A2058 cells using one-way ANOVA with Tukey’s multiple comparisons test. Created using Biorender^63^.

Another study was carried out to determine whether changing the antibody isotype to IgG4 might impact the activity of an ADC prepared in the same way. An antibody was engineered with the 225.28S variable regions but with a human IgG4 backbone for functional comparison with the equivalent IgG1 version. Whilst IgG1 antibodies possess a proline at position 228, which ensures that they only form interchain disulfide bonds at the hinge region, IgG4 contains a serine residue at position 228, which confers the ability to form inter-chain or intra-chain disulfide bonds in this region. The latter configuration allows wild-type IgG4 antibodies to be susceptible to Fab arm exchange, a process that results in unstable antibodies with reduced affinity and avidity for the target antigen^24,38^ (Fig. 3A, Upper Panel). Therefore, the IgG4 antibody used in this study contained the hinge-stabilising S228P mutation (represented by an asterisk in Fig. 3A, Upper Panel), which prevents Fab arm exchange and ensures a stable antibody format as required for potential therapeutic use (Fig. 3A, Lower Panel). 225.28S IgG4 was produced (Supplementary Fig. 1); SDS-PAGE confirmed the structural integrity of the antibody, and SEC analysis confirmed a high, reasonable degree of purity (~90%) with no significant aggregates (Fig. 3B).

Flow cytometry showed comparable binding of 225.28S IgG1 and 225.28S IgG4 against MDA-MB-231 and A2058, with similar *K*_D_ values (Fig. 3C). However, the internalisation rate of 225.28S IgG1 was significantly higher than 225.28S IgG4 after a 24-h incubation period (A2058: 68% internalisation for IgG1 compared to 27% for IgG4; MDA-MB-231: 85% for IgG1 compared to 48% for IgG4) (Fig. 3D).

To investigate whether the differences in internalisation translated into differences in payload delivery, both antibodies were conjugated to MMAE using the OYoLink^®^ method as previously described. SDS-PAGE analysis showed the presence of bands of higher molecular weight compared to the unconjugated antibody, indicating that ADC formation had occurred for both the IgG1 and IgG4 formats (Fig. 3E). 225.28S IgG1, 225.28S IgG4, and Control IgG1 in both naked antibody and MMAE-conjugated ADC formats were evaluated. Free MMAE at 30 nM reduced cell viability of MDA-MB-231 and A2058 cell lines. Treatment with 225.28S IgG1-MMAE resulted in a greater loss of cellular viability than with 225.28S IgG4-MMAE in both MDA-MB-231 and A2058 cell lines at 30 nM (Fig. 3E). Neither the unconjugated antibodies nor Control IgG1-MMAE restricted cell growth for the same incubation period.

Overall, 225.28S IgG1 demonstrated better internalisation and ADC cytotoxicity than 763.74 IgG1 and 9.2.27 IgG1, while the IgG1 antibody format was superior to IgG4 in terms of payload delivery in both CSPG4-expressing TNBC and melanoma cells.

### Generation and evaluation of 225.28S IgG1-DXd

**Fig. 4:**
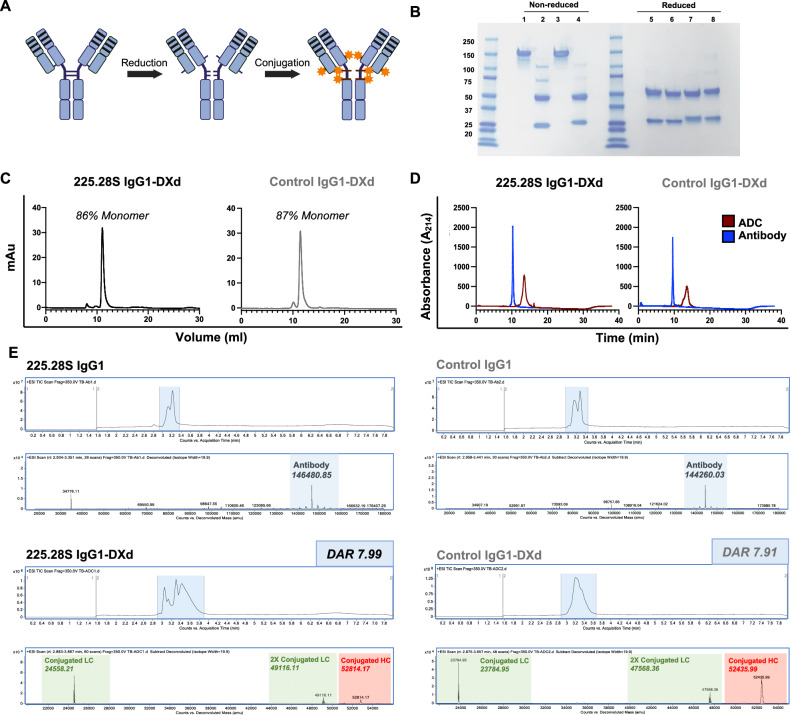
Production and characterisation of 225.28S IgG1-DXd. **A** Schematic representation of the conjugation method. 225.28S IgG1 is initially reduced with TCEP to disrupt the interchain disulfide bonds. Then, reaction of cysteine residues with deruxtecan linker-payload produces the ADC with a DAR of 8. Created using Biorender^63^. **B** SDS-PAGE characterisation of antibody and ADC. Lane 1. 225.28S IgG1 in non-reducing conditions at 150 kDa. Lane 2. 225.28S IgG1-Dxd in non-reducing conditions showing bands for heavy and light chains at 50 kDa and 25 kDa (as all interchain disulfide bonds are disrupted). Lane 3. Control IgG1 in non-reducing conditions at 150 kDa. Lane 4. Control IgG1-DXd showing bands at 50 kDa and 25 kDa in non-reducing conditions (as all interchain disulfide bonds are disrupted). Lanes 5–8: 225.28S IgG1, 225.28S IgG1-DXd, Control IgG1 and Control IgG1-DXd in reducing conditions, showing bands at 50 kDa and 25 kDa. **C** Size-exclusion chromatography (SEC) traces for 225.28S IgG1-DXd and Control IgG1-DXd. **D** Hydrophobic interaction chromatography (HIC) traces for 225.28S IgG1-DXd and Control IgG1-DXd. ADC shown in red and unconjugated antibody shown in blue. **E** Mass spectrometry (MS) analysis of antibodies conjugated to DXd. Total ion chromatography (TIC) traces and deconvoluted MS traces for antibodies and ADCs. Antibodies and ADCs were treated with PNGase F to remove antibody glycans prior to mass spectrometry analysis, and the enzyme is sometimes detected as an additional peak in the TIC traces. DAR values were calculated for Anti-CSPG4 IgG1-DXd and Control IgG1-DXd.

Given that 225.28S IgG1 had the most favourable properties for ADC development, it was next conjugated to the topoisomerase I inhibitor DXd via the thiol-maleimide reaction. This payload was chosen as it serves as the cytotoxic agent for clinically approved ADCs against HER2-positive and hormone receptor-positive breast cancers, Enhertu^®^ and Datroway^®^. To generate the targeted ADC, along with the equivalent non-targeted control ADC, both antibodies were conjugated to deruxtecan by initially fully reducing them with TCEP, followed by incubation with deruxtecan in the presence of organic solvent (Fig. 4A). SDS-PAGE analysis revealed bands at 150 kDa denoting the parent antibody in non-reducing conditions. However, in reducing conditions, bands were observed at 50 kDa and 25 kDa, corresponding to the heavy and light chains, respectively (Fig. 4B). This was consistent with conjugation products wherein the interchain disulfide bonds were reduced and conjugated with a thiol-reactive maleimide-based linker-payload to yield a DAR of 8.

SEC traces of each ADC showed monomeric products with minimal aggregation, and each ADC product comprising >85% monomer (Fig. 4C). HIC analysis showed that each ADC had a shift to a single peak at longer retention time compared to the unconjugated antibodies, also consistent with a homogeneous ADC (Fig. 4D). This was further confirmed through mass spectrometry studies. All antibody and ADC samples were first treated with peptide: *N*-glycosidase F (PNGase F) to remove heterogeneous antibody glycans before they were analysed by LC-MS, meaning that PNGase F may be present in antibody and/or ADC LC-MS traces as a result. First, the unconjugated antibodies were analysed by LC-MS to provide reference molecular weight values. Next, the ADCs were analysed by LC-MS, which showed two major peaks representing the conjugated light and heavy antibody chains. The four native interchain disulfide bonds of the parent IgG1 antibodies had been reacted with deruxtecan, resulting in the ADC constructs appearing as light and heavy chain conjugates. The weak non-covalent interactions that maintain the ADCs’ higher-order structures in the natural environment were disrupted by the denaturing mass spectrometry conditions. However, in the case of parent unconjugated antibodies, the covalent interchain disulfide bonds ensured the linkage between light and heavy chains remained intact, which appeared as intact antibodies on the mass spectrum. By making the reasonable assumption that the ADCs were composed of two light chain fragments and two heavy chain fragments, the molecular weight of each ADC was determined (Fig. 4E). By comparing the mass of ADCs and the unconjugated antibodies, a DAR of ~8 was determined. These results confirmed the successful conjugation of a homogeneous DAR 8 ADC.

### 225.28S IgG1-DXd restricts CSPG4-expressing TNBC cell viability in vitro

**Fig. 5:**
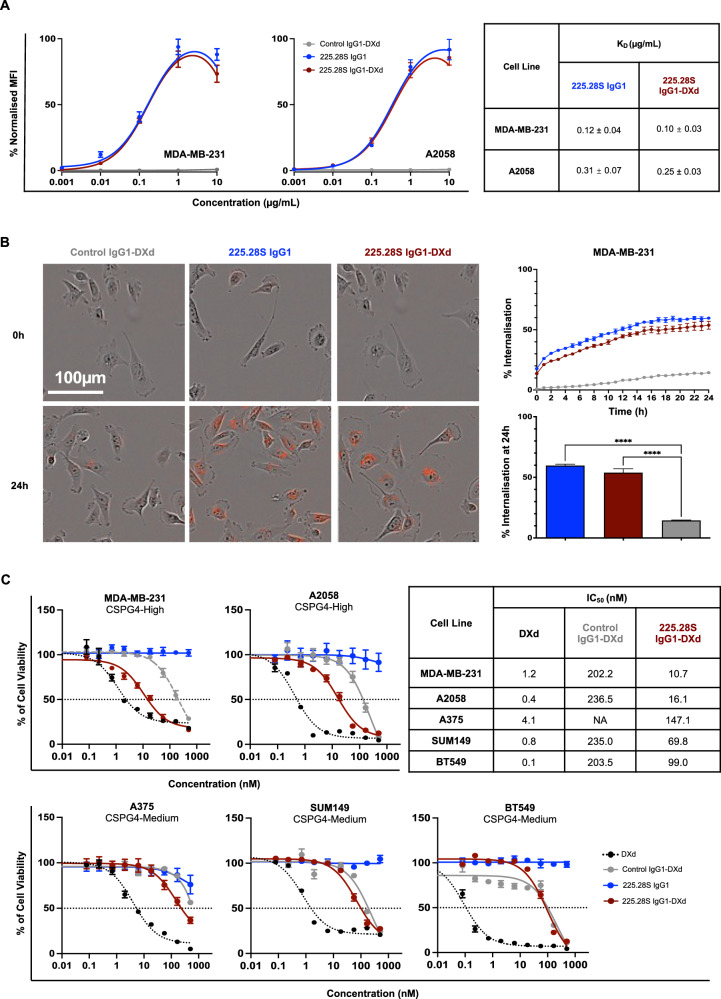
In vitro evaluation of 225.28S IgG1-DXd against TNBC. **A** Flow cytometric investigation of binding of 225.28S IgG1, 225.28S IgG1-DXd and Control IgG1-DXd to MDA-MB-231 TNBC and A2058 melanoma cell lines. MFI values are normalised to the maximum MFI value for each experiment. **B** Investigation of internalisation of FabFluor^®^-labelled 225.28S IgG1, 225.28S IgG1-DXd and Control IgG1-DXd into MDA-MB-231 cells over 24 h. Representative images and quantitation of fluorescence after 24-h incubation of cells treated with 30 nM of labelled antibody. Internalisation after 24 h compared using one-way ANOVA with Tukey’s multiple comparisons test. **C** Cytotoxicity comparison of 225.28S IgG1-DXd, 225.28S IgG1, Control IgG1-DXd, and the unconjugated Dxd payload in TNBC and melanoma cell lines after 120 h incubation.

Next, 225.28S IgG1-DXd was compared with the parent antibody, Control IgG1-DXd, and free DXd payload in functional in vitro and in vivo experiments. Flow cytometric analyses showed comparable binding of 225.28S IgG1-DXd and 225.28S IgG1 to MDA-MB-231 and A2058 cells (Fig. 5A). Similarly, 225.28S IgG1 and 225.28S IgG1-DXd exhibited comparable internalisation intensities and entry kinetics into MDA-MB-231 cells over 24 h, whilst Control IgG1-DXd showed low internalisation (Fig. 5B). CSPG4-expressing TNBC and melanoma cell lines showed sensitivity to free DXd payload, through a reduction in cell viability to <20% of PBS-treated controls after 120-h incubation. 225.28S IgG1-DXd showed robust cytotoxicity against the high CSPG4-cell lines, exhibiting IC_50_ values of 10.7 nM and 16.1 nM in MDA-MB-231 and A2058 cell lines, respectively (Fig. 5C). In the same cell lines, Control IgG1-DXd showed activity only at high doses (IC_50_ values of 202.2 nM for MDA-MB-231 and 236.5 nM for A2058), potentially due to non-specific cellular uptake, consistent with low levels of internalisation (Fig. 5B). In the medium CSPG4-expressing A375 cells and lower CSPG4-expressing SUM149 and BT549 cells, 225.28S IgG1-DXd and Control IgG1-DXd exhibited low levels of cytotoxicity only at higher doses (Fig. 5C). Taken together, these data suggest that conjugation of 225.28S IgG1 to deruxtecan does not impair target binding or internalisation and yields an effective ADC.

### 225.28S IgG1-DXd restricts growth of orthotopically grown, CSPG4-expressing PDX models of TNBC

**Fig. 6:**
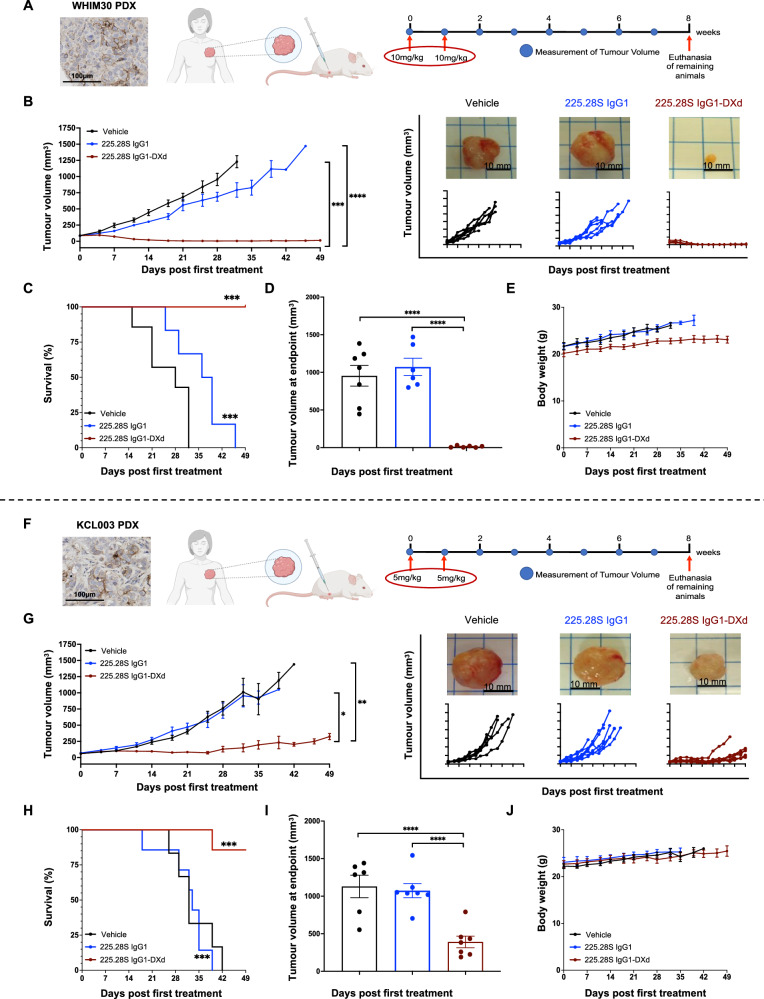
In vivo evaluation of 225.28S IgG1-DXd against different (WHIM30 and KCL003) TNBC PDX models. **A** Experimental design of WHIM30 PDX experiment. Representative image of IHC staining of WHIM30 PDX model for CSPG4 expression. Schematic representation of TNBC PDX engraftment into the mammary fat pad. Timeline of WHIM30 PDX experiment. Vehicle, 225.28S IgG1, or 225.28S IgG1-DXd were administered at 10 mg/kg on day 0 and day 7, once tumour diameters reached 50 mm^3^. Created using Biorender^63^. **B** Tumour volume measurement (left panel) for mice treated with vehicle (black), 225.28S IgG1 (blue), and 225.28S IgG1-DXd (red). Statistical analysis completed using one-way ANOVA. Tumour growth curves for individual mice, and representative images of tumours at endpoint (right panel). **C** Overall survival analysis: the Gehan-Breslow-Wilcoxon test was used for statistical analysis against the ADC group. **D** Tumour volume comparison for the dissected tumours at endpoint, defined as the time at which tumours reached 14 mm in diameter or the end of the study. Student’s *t*-test was used for statistical analysis. **E** Body weight measurements throughout the experiment. **F** Experimental design of KCL003 PDX experiment. Representative image of IHC staining of KCL003 PDX model for CSPG4 expression. Schematic representation of TNBC PDX engraftment into the mammary fat pad. Timeline of KCL003 PDX experiment. Vehicle, 225.28S IgG1, or 225.28S IgG1-DXd were administered at 5 mg/kg on day 0 and day 7, once tumour diameters reached 50 mm^3^. Created using Biorender^63^. **G** Tumour volume measurement (left panel) for mice treated with vehicle (black), 225.28S IgG1 (blue), and 225.28S IgG1-DXd (red). Statistical analysis completed using one-way ANOVA. Tumour growth curves for individual mice, and representative images of tumours at endpoint (right panel). **H** Overall survival analysis: the Gehan-Breslow-Wilcoxon test was used for statistical analysis against the ADC group. **I** Tumour volume comparison for the dissected tumours at endpoint, defined as the time at which tumours reached 14 mm in diameter, or the end of the study. Student’s *t*-test was used for statistical analysis. **J** Body weight measurements throughout the experiment.

We investigated the therapeutic potential of 225.28S IgG1-DXd in vivo in two CSPG4-expressing TNBC PDX models, whereas tumour cells were engrafted orthotopically into the mammary fat pads of immunodeficient NSG^®^ mice. Based on efficacious dosages reported in the literature for the other five deruxtecan-conjugated ADCs^39–43^, 10 mg/kg of treatments were administered to the WHIM30 PDX when tumours reached 50 mm^3^ (Fig. 6A). 225.28S IgG1-DXd significantly restricted tumour growth, with an average tumour volume of 25.2 mm^3^ at the experimental endpoint. By comparison, all the mice in the vehicle and other antibody groups reached the maximum tumour size (14 mm in diameter) before endpoint (Fig. 6B). Overall survival of the ADC-treated mice was significantly longer than mice treated with vehicle or antibody (Fig. 6C), and the dissected tumours exhibited significantly smaller volumes compared to any other group (Fig. 6D). The ADC-treated mice maintained consistent increases in body weight throughout the experiment, showing no signs of overt toxicity despite exhibiting marginally lower overall weight measurements compared to the vehicle or antibody groups (Fig. 6E and Supplementary Fig. 3).

As 10 mg/kg ADC dosing resulted in maximal tumour growth inhibition of WHIM30 xenografts, we next sought to determine whether the antitumour activity could be retained in a second TNBC PDX model, at the lower dose of 5 mg/kg to assess tumour growth restricting effects and to reduce the potential risk of any weight loss (Fig. 6F). Under the new dosing regimen, 225.28S IgG1-DXd significantly restricted the growth of the KCL003 PDX (Fig. 6G), with significantly longer overall survival (Fig. 6H), and smaller volumes of resected tumours at endpoint in the ADC group (Fig. 6I), compared to vehicle or unconjugated antibody groups. No significant changes in body weight or signs of overt toxicity were observed in any treatment groups (Fig. 6J and Supplementary Fig. 4A). Furthermore, flow cytometric assessment of CSPG4 expression on single cells extracted from tumours revealed similar CSPG4 expression levels in antibody- and vehicle-treated groups, and a significant relative reduction of CSPG4 expression of ADC-treated tumours (Supplementary Fig. 4B), consistent with the anti-tumour function of a CSPG4-targeted agent.

Taken together, these data demonstrated that a DXd-conjugated, CSPG4-targeting ADC could restrict the growth of two TNBC PDX models, without inducing any signs of overt toxicity in the mice.

### Engagement of immune cells by 225.28S IgG1 to restrict growth of TNBC tumours in vivo

**Fig. 7:**
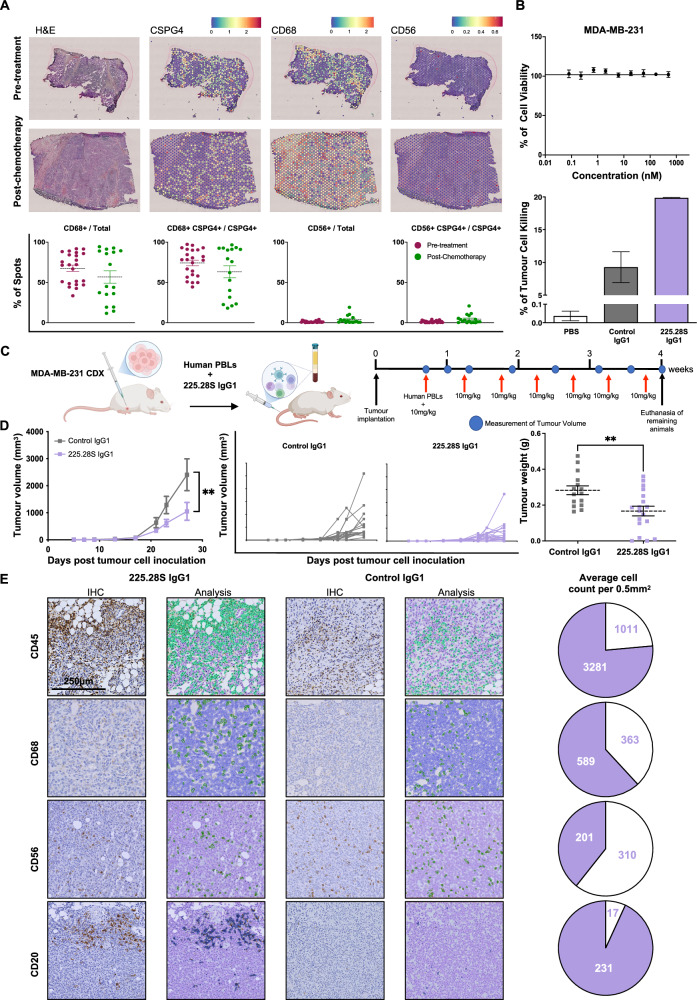
Evaluation of immune-mediated tumour growth restriction of 225.28S IgG1 against TNBC. **A** Investigation of the proportion of EpCAM-positive tumour areas also positive for CSPG4, CD68 or CD56 expression in treatment-naïve TNBC samples (*n* = 23) and residual tumour (*n* = 17) samples following treatment with neoadjuvant chemotherapy. Representative images of tumour sections. Histograms showing proportion of CD68+ or CD56+ spots out of all spots, pre-and post-chemotherapy, and the proportion of CD56+CSPG4+ or CD68+CSPG4+ spots of all CSPG4+ spots in untreated and chemotherapy-treated samples. **B** Cytotoxicity of anti-CSPG4 clones against MDA-MB-231 cells. Upper panel: 225.28S IgG1, does not restrict growth of MDA-MB-231 cells in the absence of immune cells. Lower panel: 225.28S IgG1 exhibits immune cell-mediated cytotoxicity against MDA-MB-231 cells. Data represent two independent experiments. **C** Experimental design of MDA-MB-231 CDX experiment. NSG^®^ mice implanted with MDA-MB-231 tumours were treated with 10 mg/kg of 225.28S IgG1 or Control IgG1 twice weekly. Created using Biorender^63^. **D** In vivo evaluation of 225.28S IgG1 restriction of MDA-MB-231 cell line-derived tumours. Tumour volumes from each group and for individual animals are shown. Tumour volumes on day 27 compared using the Mann–Whitney test. Weights of resected tumours from each treatment group are shown (right). Tumour weights were compared using a two-tailed, unpaired *t*-test. **E** Immunohistochemical investigation of immune cell populations in 225.28S IgG1 and Control IgG1-treated tumours. Representative images showing counts of CD45+, CD68+, CD56+, and CD20+ cells in MDA-MB-231 tumours. Average cell counts per 0.5 mm^2^ of CD45+, CD68+, CD45+ and CD20+ cells in tumours treated with 225.28S IgG1 (purple) or Control IgG1 (white), represented in pie chart. Scale bars represent 250 µm.

The next step was to identify whether 225.28S IgG1 could also be employed as a non-conjugated antibody therapeutic to engage the immune system to kill TNBC cells. First, we established whether suitable immune effector cells were present in the tumour microenvironment for the antibody to engage with. This was assessed using spatial transcriptomics analyses of untreated (*n* = 23) and NAC-treated (*n* = 17) TNBC tumours^28^ to detect expression of macrophages and NK cell markers, CD68 and CD56, respectively. Across the treatment-naïve group, the mean percentage of total CD68+ spots was 68% but was 57% across the NAC-treated samples. CD68 expression was also found to be associated with CSPG4 expression, with the average proportion of CSPG4+ spots that were also positive for CD68 being 74% in the pre-treated samples and 63% in the NAC-treated samples. No statistically significant differences between the proportion of total spots that were CD68+, or the proportion of CSPG4+ spots that were also CD68+, were observed between the pre-treated and NAC-treated cohorts (Fig. 7A). CD56+ spots, indicating the presence of NK cells, were less commonly found, representing <5% of the total spots in untreated and NAC-treated cohorts. CD56 expression was minimally associated with CSPG4 expression in both cohorts, with <5% of CSPG4+ spots being CSPG4+CD56+. Between the pre-treatment and NAC-treated cohorts, no statistically significant differences were observed in the proportion of total spots that were CD56+ or the proportion of CSPG4+ spots that were also CD56+. Together, these findings indicate that CD68+ macrophages are present in CSPG4-expressing TNBC tumours and are spatially proximal to CSPG4-expressing cells. Therefore, they could, in principle, be engaged by an anti-CSPG4 antibody to exert immune-mediated effects against CSPG4-expressing cancer cells. In vitro, while 225.28S IgG1 did not restrict MDA-MB-231 TNBC cell viability in the absence of immune cells (Fig. 7B, upper panel), the antibody exhibited immune effector cell-mediated tumour cell cytotoxicity above the level observed for the isotype control antibody when administered with human peripheral blood immune cells (Fig. 7B, lower panel).

To investigate anti-tumour cytotoxic effects in vivo, female NSG^®^ mice challenged in the mammary fat pads with MDA-MB-231 TNBC xenografts were engrafted with human peripheral blood lymphocytes (PBLs) and treated twice per week with 10 mg/kg 225.28S IgG1 or Control IgG1 (Fig. 7C). 225.28S IgG1 significantly reduced the growth of MDA-MB-231 tumour xenografts and resected tumour weight compared to Control IgG1 (Fig. 7D). Immunohistochemical analysis showed infiltration of CD45+CD68+ and CD20+ cells in the tumours treated with 225.28S IgG1 (Fig. 7E).

In summary, anti-CSPG4 antibody treatment can recruit immune effector cells into the tumour microenvironment and exert additional immunological functions against TNBC.

## Discussion

TNBC lacks effective targeted therapies, with Trodelvy the only ADC approved for its treatment^44^. The development of novel targeted therapies for this disease requires selection of tumour-associated antigens that can subsequently be targeted. We provide evidence that CSPG4 may serve as such an antigen for antibody or ADC therapies. First, we characterise expression of CSPG4 in TNBC, especially in post-NAC residual tumours, and screen anti-CSPG4 antibodies to select a suitable isotype and clone combination for potential therapeutic development. The most suitable antibody scaffold identified was then conjugated to deruxtecan to provide an experimental ADC which could restrict the growth of different patient-derived TNBC xenografts in vivo. The unconjugated antibody in the presence of immune cells was also active in vivo. Together, these findings provide the basis for the potential development of new therapies to treat TNBC.

Immunohistochemical analyses in a cohort of TNBC patient tumours showed that approximately 34% of the samples exhibited positive staining for CSPG4, adding to data provided by few previous studies which have evaluated the frequency of CSPG4 positivity across TNBC patient samples^18,19^. Furthermore, using spatial transcriptomics, we demonstrate CSPG4 expression in a cohort of treatment-naïve TNBC tumours, and retained expression in TNBC tumours treated with neoadjuvant chemotherapy, for which further treatment options are lacking. While studies in additional patient cohorts will be required to fully confirm the retention of CSPG4 expression in NAC-treated TNBC, this finding is significant as it suggests that CSPG4-specific therapies could be administered to NAC-treated TNBC patients to eradicate chemotherapy-resistant residual tumours.

Three anti-CSPG4 antibody variable regions derived from the literature were generated in a human IgG1 backbone and functionally compared to identify the best clone for ADC development. 225.28S IgG1, containing the variable regions of antibody clone 225.28S, showed superior internalisation into and cytotoxicity of cancer cells when conjugated to MMAE compared to the IgG1 antibodies with variable regions from antibody clones 763.74 and 9.2.27, respectively. These findings evidenced that the binding epitope of an anti-CSPG4 antibody can influence ADC-relevant functions, such as internalisation into target cells. These differences could be due to the specific domains to which each clone binds. The 225.28S clone variable regions have been characterised as binding to the D3 domain of CSPG4 which is also the location of two putative cleavage sites for proteases^13,45^. The 763.74 variable regions bind to the D2 domain, and the binding site of the 9.2.27 variable regions is undetermined^33,34^. If 225.28S IgG1 binds to an epitope on CSPG4 that is retained when CSPG4 is cleaved by proteases, whereas the other clones bind to sites which are removed when CSPG4 is cleaved by proteases, this could potentially explain the superior internalisation of 225.28S IgG1.

Comparisons of antibodies with the 225.28S variable regions, in IgG1 and IgG4 formats, revealed that the IgG1 exhibited superior internalisation and cytotoxicity as an ADC than the IgG4 equivalent, against CSPG4-expressing TNBC and melanoma cell lines. IgG1 and IgG4 antibodies differ in the structure of their hinge regions, and sequence differences in the lower hinge and CH2 regions contribute towards an IgG1 antibody’s superior ability to engage immune effector cells compared with IgG4 antibodies^24,25^. These structural variations may contribute towards the functional differences in the rate of cellular internalisation of anti-CSPG4 IgG1 and IgG4 antibodies. An influence of structure on the function of IgG antibodies has been previously reported by studies involving grafting of the hinge region, or the CH1 and hinge regions, between different isotypes which was shown to affect the immune stimulation exerted by agonistic antibodies^46,47^, along with antibody-dependent intracellular viral neutralisation^48^. Also, the predominance of the IgG1 format among the clinically approved ADCs suggests that it may be a superior isotype for ADC development. However, further support for this conclusion requires direct functional comparisons between ADCs comprising antibodies of different isotypes against the same epitope, and identification of the structural determinants of each isotype that may confer superior ADC-relevant functions.

225.28S IgG1 was successfully conjugated to deruxtecan, yielding a near homogeneous ADC with DAR 8. The ADC exhibited cytotoxicity against TNBC and melanoma cells, consistent with their CSPG4 cell-surface expression levels, and sensitivity to DXd payload and exhibited activity against two CSPG4-expressing TNBC PDX models in vivo, at 10 mg/kg and at lower 5 mg/kg doses. Notably, this represents the first reported example of an anti-CSPG4 antibody conjugated to the topoisomerase I inhibitor DXd, thereby expanding the therapeutic landscape of CSPG4-directed ADC strategies. Deruxtecan is the linker-payload moiety composed of a DXd and a hydrophilic cleavable tetrapeptide (GGFG) linker that reduces its overall hydrophobicity^43^. This reduces propensity for aggregation both during the conjugation process and during ADC use and storage, thereby helping to prevent the immunogenicity, rapid ADC clearance, and off-tumour toxicity associated with non-specific uptake of ADC aggregates^49^. Deruxtecan was first employed in the FDA-approved ADC Enhertu^®^ and is used in the recently approved Datroway^®^. The DXd payload is widely regarded as best in class and is employed by ADCs in regulatory review such as the anti-HER3 ADC patritumab deruxtecan (U3-1402)^40^. DXd-conjugated ADCs are also in clinical development, such as raludotatug deruxtecan which targets cadherin 6 and is being evaluated for the treatment of ovarian cancer, and ifinatamab deruxtecan which targets B7-H3 and is being evaluated for the treatment of lung and oesophageal cancers^50^. Although 225.28S IgG1-DXd exhibits a high DAR of 8, its IC_50_ values are higher than those of free DXd. A similar trend was observed for MMAE, with free MMAE demonstrating greater cytotoxicity than its corresponding ADCs under equivalent conditions. These observations are consistent with the profile of cytotoxic payloads such as DXd and MMAE that are small, membrane-permeable, and capable of rapid, target-independent cellular entry^51,52^. In contrast, ADC-mediated cytotoxicity requires antigen-specific binding, internalisation, and lysosomal processing, reflecting the trade-off between maximal in vitro potency and the reduction of off-target toxicity that underpins the therapeutic mechanism of target antigen-specific internalising ADCs.

We previously demonstrated that 225.28S IgG1 restricts the growth of melanoma xenografts in vivo in the presence of human immune cells^53^. The results presented here further show that this antibody can restrict the growth of orthotopically implanted TNBC tumours in vivo through engagement of human immune cells. These highlight antibody Fc-mediated effector functions in a potential anti-CSPG4 antibody treatment for TNBC. Evaluating immune activities of anti-CSPG4 antibodies in immunocompetent systems may be undertaken in future work. This may include transgenic mice expressing human CSPG4 and human Fcγ receptors, given that FcγR phenotypes and distribution differ significantly between humans and mice. These may provide more physiologically relevant assessments of Fc-dependent immune activation and potential on-target as well as Fc-mediated toxicities. Alongside this work, further pharmacokinetic, maximum tolerated dose and organ toxicity, assessments would enable a deeper investigation into the safety profiles and immune mechanisms underlying the functions of anti-CSPG4 antibodies and ADCs, supporting their translation.

**Fig. 8:**
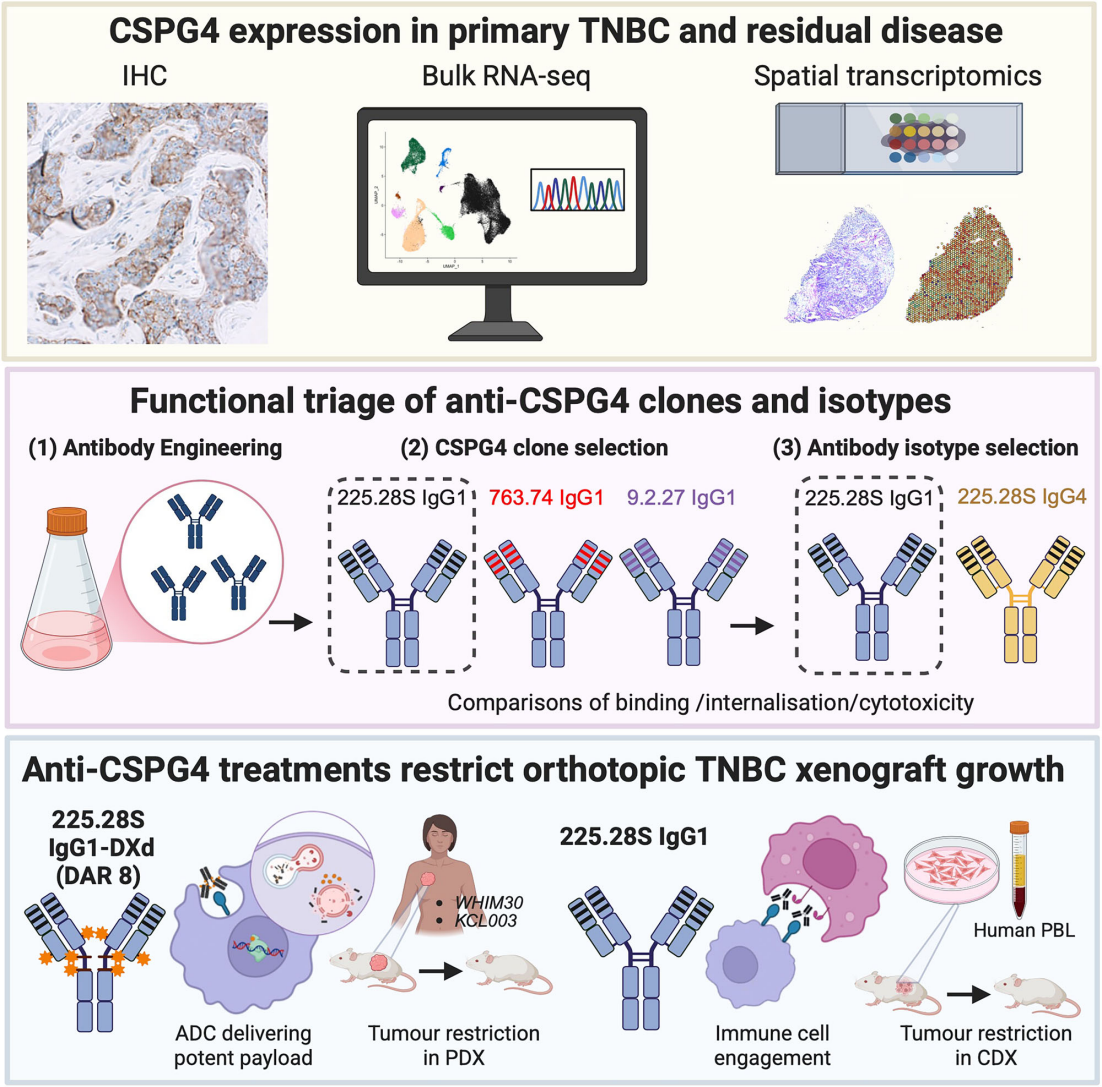
Schematic representation of investigation of CSPG4 expression in TNBC, functional triage of antibody scaffolds, and evaluation of anti-CSPG4 antibodies and ADCs in vitro and in vivo. Created using BioRender^63^.

In conclusion, the in vitro and in vivo activity of the 225.28S IgG1, in both its naked form and as an ADC conjugated to deruxtecan, suggest that CSPG4-targeted therapies could be developed as effective novel treatments for CSPG4-expressing TNBC. Our study also highlights the importance of considering antibody clone and isotype for ADC development (Fig. 8).

## Methods

### Gene expression analyses of CSPG4 in breast cancers

Gene expression analysis of CSPG4 expression in breast cancer was carried out using bulk and single cell RNA-seq (scRNA-seq), and spatial transcriptomics analyses. Detailed descriptions of different transcriptomic datasets analysed in this study have been published previously: the TNBC-enriched King’s College London Guy’s Hospital cohort (Guy’s) (*n* = 177)^54^, The Sweden Cancerome Analysis Network (SCAN-B) (*n* = 3273)^55,56^, Molecular Taxonomy of Breast Cancer International Consortium (METABRIC) (n = 1380)^57,58^, and The Cancer Genome Atlas (TCGA) (RRID:SCR_003193) (*n* = 1084)^59^, and the International Cancer Genome Consortium (ICGC) cohort (*n* = 259)^60^. Clinicopathological and gene expression data were extracted and compared between IHC-defined subtypes based on breast pathology evaluation of ER, PR, and HER2 receptors, or between PAM50 subtypes based on the expression of 50 genes to subclassify breast cancers into five distinct subtypes (i.e., Basal-like, HER2-enriched, Luminal A, Luminal B, and Normal-like breast cancer). PAM50 information was not included in the ICGC cohort. Statistical analyses and data plots were generated in R version 4.2.2.

Bulk and single-cell RNA-sequencing (scRNA-seq) analysis of expression of CSPG4 and other molecular and immune markers in breast cancers was carried out using data obtained from a publicly available dataset (GSE161529) using pre-processed data objects produced by the authors^27^. Individual Seurat objects SeuratObject_HER2.rds, SeuratObject_ERTotal.rds and SeuratObject_TNBC.rds were downloaded from Figshare and read into R using the readRDS function. Uniform Manifold Approximation and Projection (UMAP) visualisation for all objects were generated using the RunUMAP function in Seurat (v5.1.0) (RRID:SCR_007322) using 20 dimensions as input features. TNBC, ER+ and HER2+ single-cell datasets were integrated, and batch corrected to allow a breast cancer subtype comparison. The SelectIntegrationFeatures function was used to find the most variable features across samples to integrate and was set to return 3000 features. The objects were then merged using the merge function and then split based on breast cancer patient samples. The NormaliseData function was used to normalise the object.

Spatial analysis of CSPG4 and other markers in TNBC samples was investigated from a publicly available dataset (GSE210616). Seurat objects for each patient slice were normalised using SCTransform, and individual genes of interest were mapped onto each Seurat object using the SpatialFeaturePlot function. Count data was used to quantify gene expression per sample and per patient, and to quantify the number of double positive spots.

The Km Plotter platform (RRID:SCR_018753) was used to investigate expression of CSPG4 at the transcriptomic and protein level, and its relationship to survival of TNBC patients.

### **Immunohistochem**ical analyses of CSPG4 in breast cancers

CSPG4 immunohistochemical staining was performed on primary breast tumour tissue samples that had been previously characterised^61^. Formalin-fixed paraffin embedded (FFPE) Tissue Microarrays (TMAs) were constructed from patient tumour samples, mainly from the periphery of the carcinoma and other representative areas of the invasive tumour. CSPG4 expression was assessed on 3 μm thick TMA and cell pellet sections, stained using an automated VENTANA (Roche Diagnostics) platform with an ultra-view universal DAB Detection kit, followed by a haematoxylin counterstain. IHC was performed using Anti-CSPG4 (1:100) Rabbit Polyclonal (Atlas Antibodies, Cat# HPA002951, RRID: AB_1854449) with CC1/CC2 tissue pre-treatment for 64 min at 95 °C. A blank control was used for detection of non-specific background staining.

A375 human melanoma cells served as a positive control. Detection of a combination of cytoplasmic and membrane staining was considered as positive for CSPG4. When specific staining was not detected, the result was considered negative. Analyses were performed using digital images obtained by the NanoZoomer HT Digital Pathology Scanning System (Hamamatsu).

### Antibody engineering and production

225.28S IgG1, comprising the variable regions of the murine clone 225.28S in a human IgG1 format, was produced as previously described^17^. Production of plasmid vector encoding the anti-CSPG4 antibody based on the murine clone 225.28S^18^ in an IgG4 backbone was carried out as described previously^35^, to generate a pVitro1-hygro-mcs plasmid (InvivoGen) encoding 225.28S IgG4. The variable regions of the anti-CSPG4 antibodies 763.74^29^ and 9.2.27^30^ and the isotype control antibody against 4-hydroxy-3-iodo-5-nitrophenylacetic acid (NIP) were cloned into a human IgG1 backbone as previously described^35^, to generate pVitro1 plasmids encoding 763.74, 9.2.27, and Control IgG1, respectively (Supplementary Fig. 1).

Antibody production was carried out through transient transfection of Expi293F cells (Gibco, Cat# A14527, RRID: CVCL_D615), according to the manufacturer’s instructions. After 120 h of culture, cell supernatants were harvested and centrifuged at 3000 rpm for 15 min before purification of the antibody using HiTrap Protein A 1 mL columns (Cat# 71-7002-00 AR). The antibody was loaded onto the column, washed with 10 mL of Phosphate Buffered Saline (Thermo Fisher Scientific, Cat# 10010023), eluted using 0.1 M glycine pH 3.5, and neutralised with 1 M TRIS pH 8.5. Antibody was concentrated and buffer exchanged into PBS using Amicon Ultra Centrifugal Filters with 50 kDa molecular weight cutoff (Sigma-Aldrich, Cat# UFC905024).

### ADC production and purity analysis

Antibodies were conjugated to the tubulin inhibitor monomethyl auristatin E (MMAE) using the oYo-Link® VcMMAE system (AlphaThera, Cat# AT7001) according to the manufacturer’s instructions. Unconjugated excess linker-payload was removed using oYo-Capture™ Magnetic Beads (AlphaThera, Cat# AT8101-10) according to the manufacturer’s instructions. To generate DXd-conjugated ADCs, antibody was first reduced by incubation with 30 molar equivalents of tris(2-carboxyethyl) phosphine hydrochloride (TCEP·HCl) (Sigma-Aldrich, Cat# 646547) for 2 h at 37 °C, shaking at 300 rpm. Reduced antibody was then incubated with 40 molar equivalents of the deruxtecan reagent (MedChemExpress, Cat# HY-13631E) for 3 h, shaking at 300 rpm at room temperature. The ADC was concentrated and buffer exchanged to PBS using an Amicon® Ultra Centrifugal Filter, 10 kDa molecular weight cutoff (MWCO) (Sigma-Aldrich, Cat# UFC801008).

Antibody and ADC purity was confirmed using size-exclusion chromatography (SEC) using a Superdex™ 200 10/300 GL column (Cytiva, Cat# GE28-9909-44) and an Äkta purifier system (GE Healthcare) at 4 °C. Two column volumes (CV) of PBS were subsequently run through the column and protein was detected by UV absorbance at 280 nm (A_280_). Peaks from the chromatographic run eluting before or after the monomeric peak were considered as aggregates or degradants, respectively. Peak percentages were calculated by dividing individual peak areas by the sum of peak areas on UNICORN™ 7.0 software.

Sodium dodecyl sulfate polyacrylamide gel electrophoresis (SDS-PAGE) was used to investigate antibody structural integrity. The antibody or ADC was prepared in PBS and 4X Laemmli buffer (Bio-Rad Laboratories, Cat# 1610747) and boiled at 95 °C for 5 min. For samples prepared under reducing conditions, 1 µL of β-mercaptoethanol was added per 10 µL of sample prior to boiling. The protein was then loaded onto a 4-15% Mini-P ROTEAN TGX Precast Protein Gel 10-well, 30 µL (Bio-Rad Laboratories, Cat# 4561083) with 20 µL of sample containing approximately 2 µg of antibody alongside 5 µL of the Precision Plus Protein Dual Color Standard (Bio-Rad Laboratories, Cat# 1610394). Gels were run for 45 min at 150 V at room temperature. Gels were stained and protein bands were visualised using InstantBlue^®^ Coomassie Protein Stain (Abcam, Cat# AB119211).

### ADC DAR characterisation

Hydrophobic interaction chromatography (HIC) was employed to determine the drug-antibody ratio (DAR) of conjugated ADCs. Separations were performed on a TSKgel Butyl-NPR column (4.6 × 100 mm, 2.5 μm) operated at a flow rate of 0.5 ml/min. Mobile phase A consisted of 25 mM sodium phosphate buffer (pH 7.0) containing 1.5 M ammonium sulfate, and mobile phase B was 25 mM sodium phosphate buffer (pH 7.0) mixed with 80% 2-propanol. A linear gradient from 0 to 100% B was applied over 40 min. Elution was monitored by UV detection at 214 nm. Individual DAR species were resolved based on their relative hydrophobicity.

Molecular masses of proteins ( > 40 kDa) were further measured by mass spectrometry.

All protein samples were desalted using Zebaspin (7 kDa MWCO, Thermo Fisher Scientific) prior to LC-MS analysis. The protein sample (20 µL, 5 µM) was deglycosylated with 0.4 μL PNGase (New England Biolabs) for 40 h at 8 °C prior to LC-MS submission. Two μL of a protein sample (diluted to 0.2 mg/mL in LC-MS grade water) was separated on the column using mobile phase A (water, 0.1% formic acid) and B (acetonitrile, 0.1% formic acid) with an eluting gradient (shown below) at a flow rate of 0.8 mL/min. The oven temperature was maintained at 60 °C. The mobile phase gradient used for A/B elution is provided in Supplementary Table 1. Agilent 6530 QTOF mass spectrometer was operated in a positive polarity mode, coupled with an ESI ion source. The ion source parameters were set up with a VCap of 4000 V, a gas temperature at 350 °C, a dry gas flow rate at 10 L/min and a nebuliser of 35 psi. MS TOF was acquired under conditions of a fragmentor at 350 V, a skimmer at 65 V and an acquisition rate at 1 spectra/s in a profile mode, within a scan range between 100 and 7000 m/z. The data were then analysed by deconvoluting a spectrum to a zero-charge mass spectrum using a maximum entropy deconvolution algorithm within the MassHunter software version B.07.00.

### Cell lines and culturing

Human embryonic kidney Expi293F cells were cultured in Expi293 Expression Medium (ThermoFisher Scientific, Cat# A1435101) in 125 cm Erlenmeyer cell culture flasks (Corning, Cat# CLS431143) on an orbital shaker at 125 rpm at 37 °C and 8% CO_2_. A2058, A375 and BT549 cell lines were sourced from ATCC. The HCC1187, MDA-MB-231, and SUM149 cell lines were kindly provided by the Breast Cancer Now Unit at King’s College London. The WM1361 cell line was kindly provided by Dr Vicky Sanz-Moreno (King’s College London). Cancer cell lines A2058 (RRID:CVCL_1059), A375 (RRID:CVCL_0132), MDA-MB-231 (RRID:CVCL_0062), and SUM149 (CVCL_3422) were cultured in Dulbecco’s Modified Eagle Medium (DMEM) (Thermo Fisher Scientific, Cat# 11965084). The cell lines WM1361, HCC1187 (RRID:CVCL_1247), and BT549 (RRID:CVCL_1092) were cultured in Roswell Park Memorial Institute (RPMI) 1640 Medium (Thermo Fisher Scientific, Cat# 11875093). Media was supplemented with 10% FBS and 1% Penicillin-Streptomycin (10,000 U/mL) (Thermo Fisher Scientific, Cat# 15140122). Cell lines were authenticated by short tandem repeat profiling. Cells were used only after testing negative for mycoplasma and maintained for up to 30 passages. All cell lines were cultured at 37 °C in a 5% CO_2_-humidified incubator and passaged by detachment in PBS containing 5 mM EDTA.

### Flow cytometry

For investigation of extracellular CSPG4 expression, cell lines were resuspended in FACS buffer comprising 1% FBS (Thermo Fisher Scientific, Cat# A5256801), and 2 mM EDTA (Lonza, Cat# 20356) in PBS at a concentration of 100,000 cells/mL. A total of 100 µL of cells per well were plated in Nunc™ 96-Well Polystyrene Round Bottom Microwell Plates (Thermo Fisher Scientific, Cat# 268200). After centrifugation at 300 $\times$ *g* for 5 min, supernatants were removed, and each well of cells re-suspended in 100 µL of FACS buffer containing 0.5 µg of primary antibody for investigation of surface expression, or at the relevant concentration for generation of binding curves. After incubation at 4 °C for 30 min, plates were centrifuged in the previous conditions and supernatants were removed before re-suspension of the cells in 100 µL of FACS Buffer containing Goat Anti-Human IgG-FITC (Native Antigen Company, Cat# 5230-0291) at a concentration of 2 µg/mL. After incubation for 30 min at 4 °C in the dark, cells were washed three times with 100 µL of PBS, before resuspension in 100 µL of FACS buffer and placed at 4 °C in the dark prior to data acquisition using a CytoFLEX LX instrument (Beckman Coulter, RRID:SCR_025067). For experiments comparing binding of antibodies to CSPG4 on target cells, MFI values are normalised to the maximum MFI value for each experiment.

### Internalisation studies

Cells were plated in 10% FBS FluoroBrite DMEM (Thermo Fisher Scientific, Cat# A1896701) or RPMI 1640 Medium, with no phenol red (Thermo Fisher Scientific, Cat# 11835030) at 1000 cells per well, 24 h before imaging. For experiments using Human FabFluor-pH Red Antibody Labeling Dye for Antibody Internalization (Labelling Dye) (Essen Bioscience) (Cat# 4722), 1 µL of FabFluor dye, diluted according to manufacturer instructions, was incubated with 10 µL of 300 nM antibody or ADC on ice for 30 min before addition to cells for a final concentration of 30 nM labelled antibody. Images were obtained by an Incucyte S3 system (Essen Bioscience, RRID:SCR_023147) and data analysed using the Incucyte Zoom software.

### Cell viability studies

Cell viabilities were detected using CellTiter 96 AQueous One Solution Cell Proliferation Assay (Promega, Cat# G3582) according to the manufacturer’s instructions, and measured by FLUOStar Omega Microplate Reader (BMG LabTech, RRID:SCR_025024), or by CellTiter® Glo 2.0 assay (Promega, Cat# G9242), and luminescence was measured using CLARIOstar Plus microplate reader (BMG LabTech). Free MMAE (MedChemExpress, Cat# HY-15162) and free DXd payload (MedChemExpress, Cat# HY-13631D) were included as comparators in cytotoxicity assays assessing the activity of deruxtecan- and MMAE-conjugated ADCs.

### Immune effector cell killing assay

Antibody-dependent cancer cell killing in the presence of human peripheral blood mononuclear cells (PBMCs) was carried out using flow cytometry and cell deaths were calculated as previously described^62^. In detail, cancer cells were washed in Hank’s Balanced Salt Solution (HBSS) (Thermo Fisher Scientific, Cat# 14060040) prior to co-culture with PBMCs, and incubated with 0.5 mM cell tracker carboxyfluorescein succinimidyl ester (CFSE) for 10 min at 37 °C. Cells were then washed with complete DMEM and incubated overnight in normal growth media. 50 µL of 1 × 10^6^/mL CFSE-labelled cancer cells and 50 µL of 3 × 10^6^ cells/mL PBMCs were added to a round-bottom 96-well plate in the presence of 10 µg/mL antibody and incubated for 3.5 h. Cells were washed in FACS buffer and incubated for 1 h on ice with anti-CD14-Alexa 647® antibody (BioLegend, Cat# 325612, RRID: AB_830685). Cells were then washed again and re-suspended in DAPI-containing FACS buffer, and data were acquired using a Beckman Coulter CytoFLEX instrument. Analysis was performed using the FlowJo software (RRID:SCR_008520). Healthy volunteer leucocyte cones were supplied by the UK National Health System Blood and Transplant service.

### Patient-derived xenograft (PDX) models

Tumour samples were collected from patients via surgery or biopsies with informed consent. Samples were anonymized and assigned a King’s College London (KCL) number, and the presence of tumour material was confirmed by a clinician histopathologist or pathology-trained technician. All animal experiments were approved by the KCL Institutional Committee on Animal Welfare, and in compliance with the United Kingdom Home Office Animals Scientific Procedures Act, 1986 and performed under PPL PF64A32A and PP5228677. All procedures were performed under aseptic conditions. Mice were kept under pathogen-free conditions on a 12-h light/dark cycle (light of 350–400 lux). Housing conditions were maintained at 22 °C, and at a relative humidity of 40 to 60%.

Female NSG^®^ mice (NOD SCID gamma NSG; NOD.Cg-Prkdc^SCID^Il2rg^tm1Wjl^/SzJ) were obtained commercially from Charles River Laboratories. KCL003 or WHIM30 patient tumours were dissociated to single cells and orthotopically-implanted into the mammary fat pads of NSG^®^ mice. Once tumours developed, these were serially transplanted either as single cells or tumour chunks into naïve cohorts of 3–8-week-old mice to generate KCL003 and WHIM30 PDX models.

For the ADC treatment studies, 0.1 mg/kg of Vetergesic was administered via intraperitoneal injection in 4–5 week old female NSG^®^ mice. Mice were then anaesthetised in an induction chamber using isoflurane. The fur covering the right flank of mice was shaved while mice were under anaesthesia. Tumour chunks comprising 2 mm pieces of viably frozen KCL003 or WHIM30 tumour tissue (from a single mother tumour) were implanted into the mammary fat pad using a trocar. Mice were allowed to recover from anaesthesia on a heated pad and were subsequently transferred to a clean cage heated to 37 °C until full recovery. Mice were weighed on the day of implantation and throughout the study. Tumours were measured with callipers, and volumes were calculated by the formula: π × length × width^2^/6, where length is the largest tumour diameter and width is the perpendicular diameter. Once tumours reached 50 mm^3^, mice were randomised to four experimental groups and given intravenous injection of PBS vehicle control, unconjugated 225.28S IgG1 antibody, or 225.28S IgG1-DXd (all at 10 mg/kg for WHIM30 or 5 mg/kg for KCL003). For both PDX models, each treatment group contained at least six mice. A second dose at the same concentration was given 7 days after the first injection. Mice were euthanised once tumours reached 14 mm in diameter or at experimental endpoint, by exposure to carbon dioxide gas in a rising concentration, followed by cervical dislocation. All treated animals were reported and included in the experimental and statistical analyses.

### Flow cytometric assessment of CSPG4 expression on ex vivo KCL003 PDX

To identify CSPG4 expression ex vivo, tumour chunks were crushed through a 40 µm cell strainer to extract single cells. Cell suspensions were washed three times in PBS and stained with 225.28S IgG1 for 30 min on ice, followed by a goat anti-human IgG FITC secondary antibody (2BScientific; RRID: AB_218360) for 20 min on ice. DAPI was used as the live/death marker. Samples were acquired using the FACSCanto II flow cytometer equipped with BD FACSDiva Software (BD Biosciences; RRID:SCR_001456), and data were analysed with FlowJo_V10 software (RRID:SCR_008520) to measure MFI.

### Cell line-derived xenograft (CDX) model

Single cell suspensions containing 1$\times$10^6^ MDA-MB-231 cells pre-mixed with BD Matrigel Matrix (Corning, Cat# 354277) diluted 1:1 with sterile PBS were injected orthotopically into the fat pad of 3-week-old NSG^®^ mice. In 5 days following tumour challenge, mice were injected intravenously with 1$\times$10^7^ human peripheral blood leucocytes (PBL) from healthy volunteer donors. Mice were treated with either 10 mg/kg Control IgG1 (*n* = 15) or 10 mg/kg 225.28S IgG1 (*n* = 18). A single antibody treatment was administered with the first dose of PBLs at 10 mg/kg, followed by 10 mg/kg doses administered twice a week. The animals were sacrificed 4 weeks post-tumour cell inoculation.

For identification of CD45+, CD56+, CD68+ and CD20+ cells in MDA-MB-231 cell line-derived tumours by immunohistochemistry, positive cell counts were assessed using build-in tools in QuPath 0.4.1. Cells were detected using cell detection tool followed by application of object classifier (rTrees) trained on representative regions of interest within slides to identify positive cells (DAB-stained cells) and negative cells.

For all in vivo studies, power calculation to determine sample sizes (5% significance, 90% statistical power) are based on a two-tailed, Mann–Whitney *U* test. Stratified randomisation has been performed for all efficacy studies. An independent researcher was assigned to blindly measure parameters such as tumour size to reduce or eliminate some sources of experimental bias. Animals were acclimatised for a minimum of six days prior to treatments.

### Ethics approval

Human samples were collected with informed written consent, in accordance with the Helsinki Declaration, and the study design was approved by London-Chelsea Research Ethics (REC number: 13/LO/1248, IRAS ID 131133). Patients were staged and classified according to the TNM Classification of Malignant Tumours accepted by the Union for International Cancer Control.

### Statistical analyses

Statistical analyses were performed using GraphPad Prism (RRID:SCR_002798) where three or more independent experiments were carried out. Unless otherwise stated, data were presented as the mean ± SEM values. A Shapiro–Wilk normality test was performed to evaluate normality. Subsequent appropriate statistical analyses were performed, such as two-tailed unpaired Student’s *t* test, One-way ANOVA for normally distributed data; Mann–Whitney or Kruskal–Wallis test for non-parametric data and two-way ANOVA. Statistically significant differences are indicated in the graphs or additional tables were indicated. *p < 0.05, **p < 0.01, ***p < 0.001, ****p < 0.0001.

## Supplementary information

Supplementary figures

## Data Availability

Data are available upon reasonable request from the corresponding authors. Publicly available datasets used in this study include Km Plotter https://kmplot.com/, GSE161529^27^, and GSE210616^28^.
