# Supplementary material for: An antibody-drug conjugate designed through clone and isotype selection restricts the growth of CSPG4-expressing triple-negative breast cancer
Source: NPJ Precis Oncol. 2026 Mar 7;10:161. doi: 10.1038/s41698-026-01341-0 (PMC13096323; doi:10.1038/s41698-026-01341-0)
Supplement: Supplementary file 1 — Supplementary figures [file 41698_2026_1341_MOESM1_ESM.pdf]

# SUPPLEMENTARY FIGURES

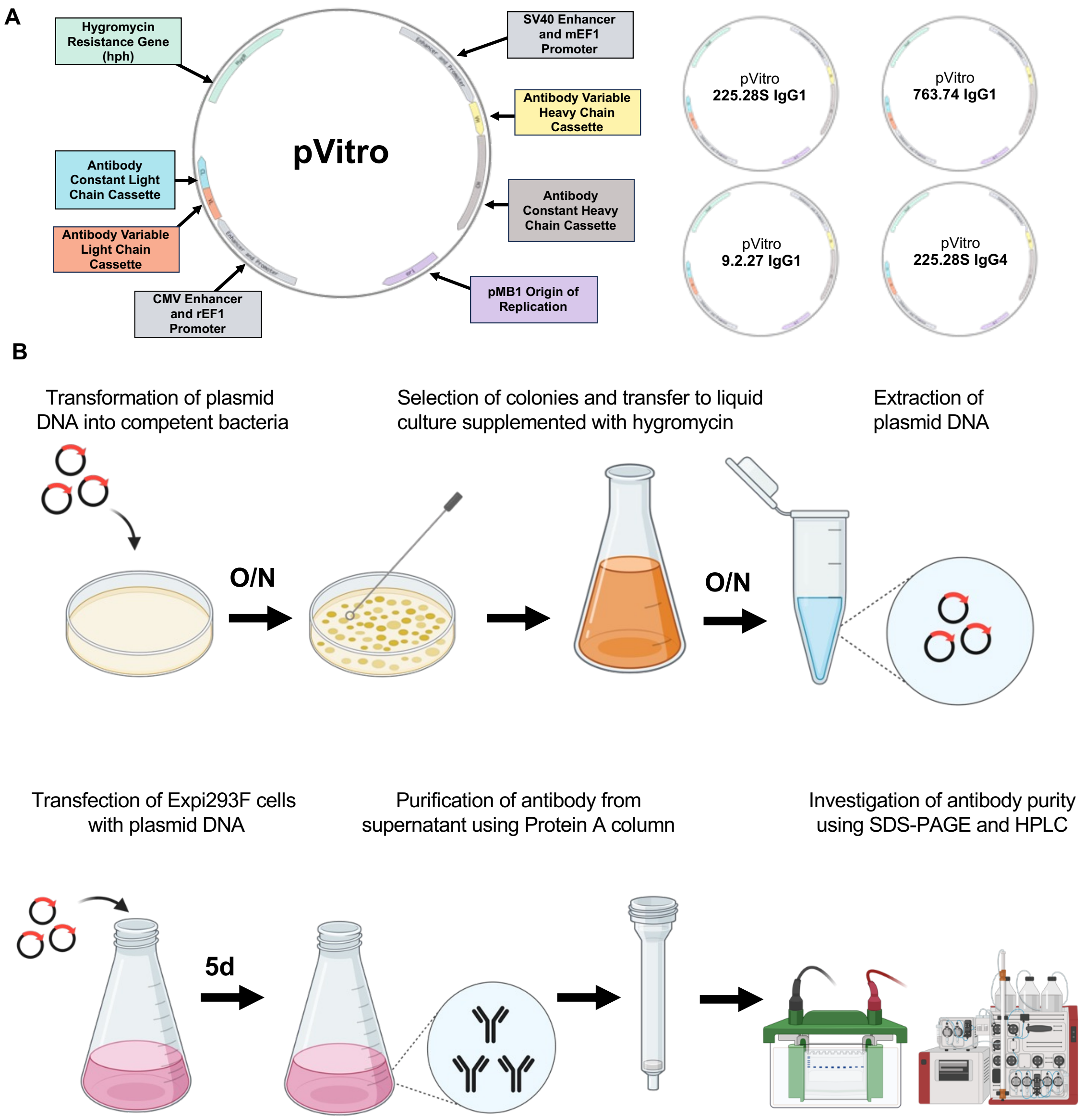


**Supplementary Figure 1. Cloning and Production of Anti-CSPG4 Antibodies.**

**A.** Schematic diagram of the features of pVITRO plasmid vector utilised for antibody engineering, and schematic representation of the source plasmids used. **B.** Schematic representation and description of the process of bacterial transformation, DNA extraction, transfection, and antibody production and characterisation (O/N – Overnight). Created using Biorender (1).

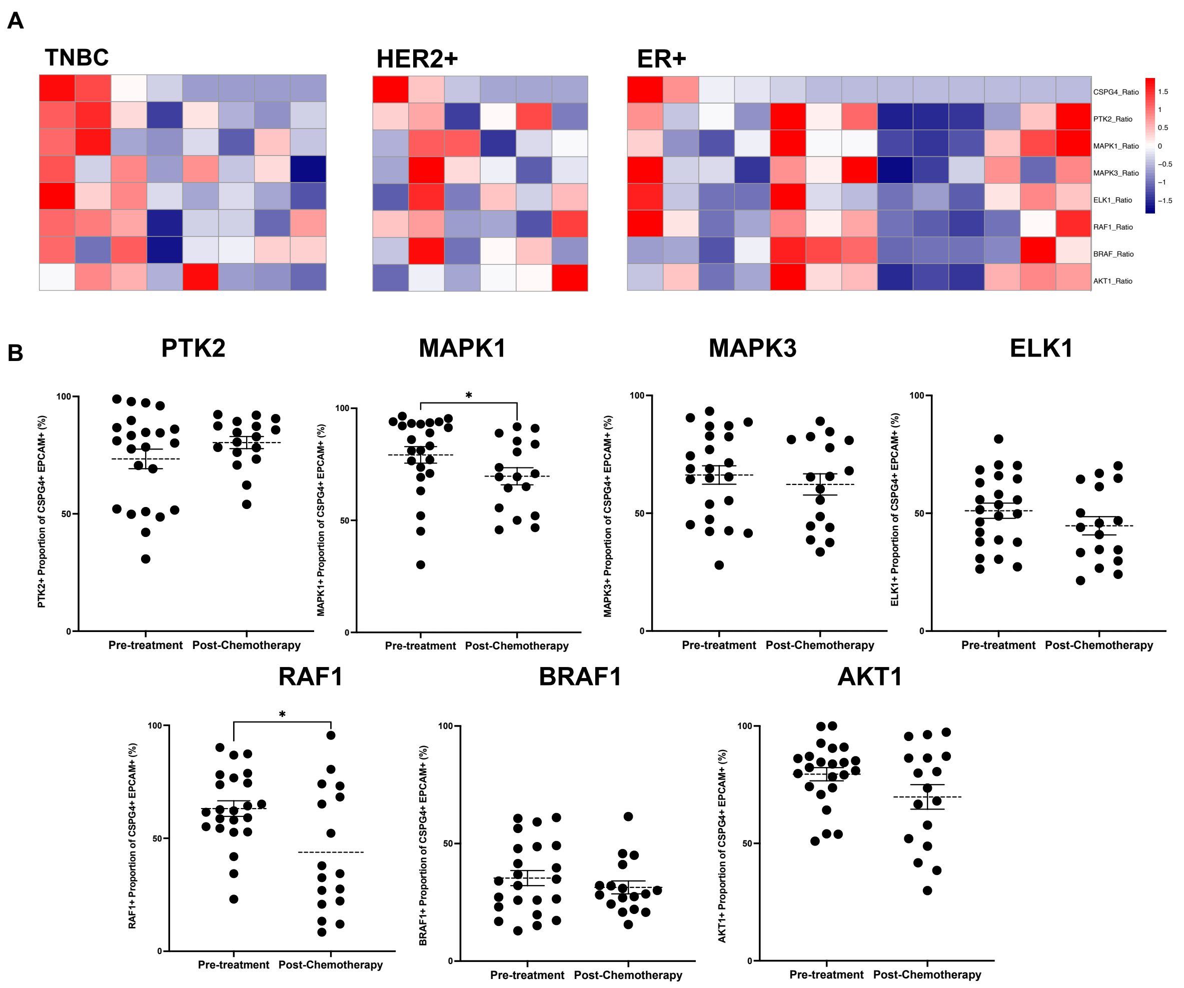


**Supplementary Figure 2: Expression of CSPG4 and Associated Signalling Pathway Molecules in Breast Cancers. A.** Expression of CSPG4, and the kinases BRAF, Raf1, MAPK1 (Erk2), MAPK3 (Erk1), and Akt1 was investigated in a scRNA-seq dataset of TNBC (n = 8 samples, 58419 cells), ER+ (n = 13 samples, 63554 cells) and HER2+ (n = 6 samples, 31917 cells) breast cancers. Each column represents a patient tumour sample. **B.** Spatial transcriptomics investigation of the expression of kinase genes in TNBC tumours pre- and post-chemotherapy. Histograms compare the proportion of CSPG4+EpCAM+ spots which are also positive for each kinase gene between untreated (n = 23) and NAC-treated (n = 17) TNBC patient cohorts. Comparisons were carried out using the Mann-Whitney test.

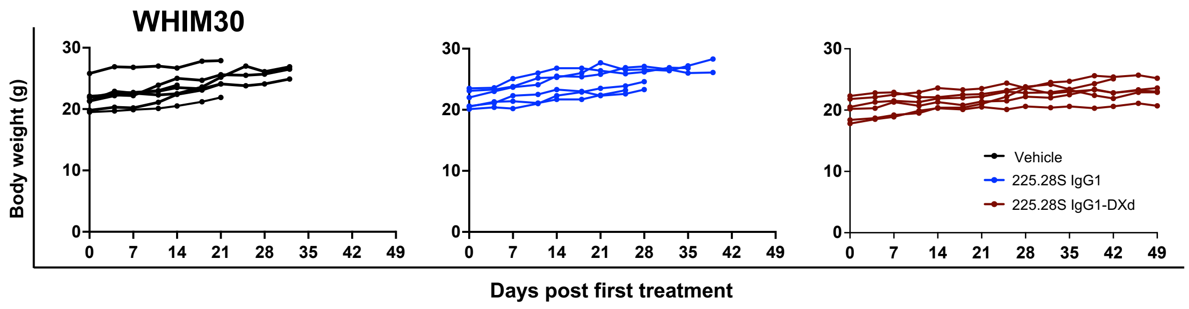


**Supplementary Figure 3. Body weight measurements of immunodeficient mice implanted with WHIM30 TNBC PDX**. Individual body weights of NSG® mice orthotopically implanted with WHIM30 PDX tumours and treated with vehicle (black), 225.28S IgG1 (blue) and 225.28S IgG1-DXd ADC (red)**.**

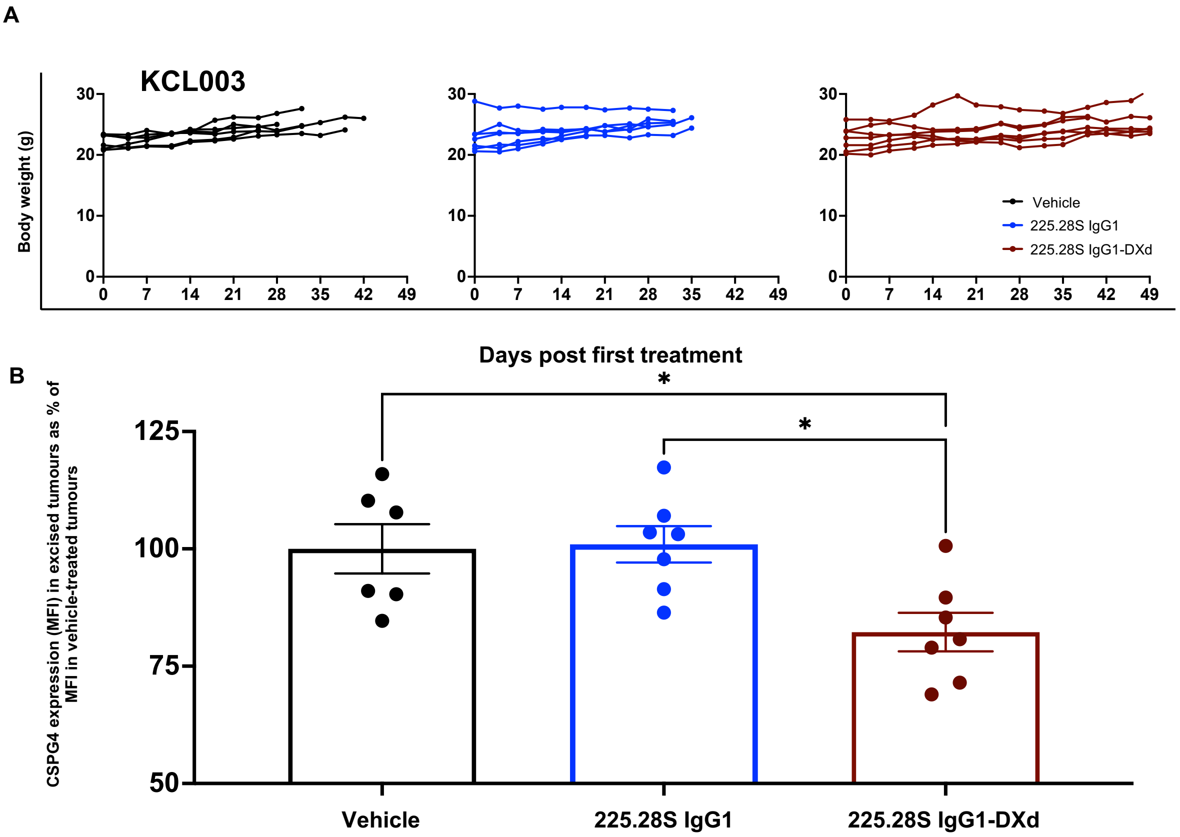


**Supplementary Figure 4.** ***In Vivo*** **Evaluation of 225.28S IgG1-DXd treatment of KCL003 TNBC PDX xenografts in immunodeficient mice**. **A.** Individual body weights of NSG® mice implanted orthotopically with KCL003 PDX tumours and treated with antibody or ADC. Vehicle (black), 225.28S IgG1 (blue), 225.28S IgG1-DXd ADC (red). **B.** Flow cytometric analysis of CSPG4 antibody target expression (MFI) in KCL003 xenograft tumours dissected at the end of the study. Bar graphs depicting relative MFI of CSPG4 expression on the surface of cells from xenografts, as a percentage of CSPG4 expression (MFI) of cells from tumours of animals given vehicle only. MFI of cells extracted tumours mice treated with vehicle (black), 225.28S IgG1 (blue), and 225.28S IgG1-DXd ADC (red). Statistical comparisons carried out using Student's T test.

## SUPPLEMENTARY TABLE

| Time (min) | Solvent A (%) | Solvent B (%) |
|---|---|---|
| 0 | 80 | 20 |
| 1 | 80 | 20 |
| 6.5 | 40 | 60 |
| 7.5 | 40 | 60 |
| 7.6 | 80 | 20 |
| 8.5 | 80 | 20 |

**Supplementary Table 1. Liquid Chromatography – Mass Spectrometry (LC-MS) mobile phase gradient for Solvent A/Solvent B elution.** The protein sample (20 µL, 5 µM) was deglycosylated with 0.4 µL PNGase (New England Biolabs) for 40 hours at 8°C prior to LC-MS submission. 2 µL of a protein sample (diluted to 0.2 mg/mL in LC-MS grade water) was separated on the column using mobile phase A (water, 0.1% formic acid) and B (acetonitrile, 0.1% formic acid) with an eluting gradient at a flow rate of 0.8 mL/min.

# REFERENCES

1. Karagiannis S. 2025. Available from: https://BioRender.com/2832qh8.

# The ARRIVE Essential 10: Compliance Questionnaire

Use this questionnaire to evaluate how well a manuscript complies with the ARRIVE Essential 10. It can be applied to any manuscript describing comparative experiments in living animals, by assessors such as journal staff, editors, or peer reviewers.

| Item | | Question(s) | Answers |
|---|---|---|---|
| 1 | Study Design | Are all experimental and control groups clearly identified? | ☒ Yes, for at least one experiment<br>☐ No |
| | | Is the experimental unit (e.g. an animal, litter or cage of animals) clearly identified? | ☒ Yes, for at least one experiment<br>☐ No |
| 2 | Sample Size | Is the exact number of experimental units in each group at the start of the study provided (e.g. in the format '*n*=')? | ☒ Yes, for at least one experiment<br>☐ No |
| | | Is the method by which the sample size was chosen explained? | ☒ Yes, for at least one experiment<br>☐ No |
| 3 | Inclusion & Exclusion Criteria | Are the criteria used for including and excluding animals, experimental units, or data points provided? | ☐ Yes, for at least one experiment<br>☒ No |
| | | Are any exclusions of animals, experimental units, or data points reported, or is there a statement indicating that there were no exclusions? | ☒ Yes, for at least one analysis<br>☐ No |
| 4 | Randomisation | Is the method by which experimental units were allocated to control and treatment groups described? | ☒ Yes, for at least one experiment<br>☐ No |
| 5 | Blinding | Is it clear whether researchers were aware of, or blinded to, the group allocation at any stage of the experiment or data analysis? | ☒ Yes, for at least one experiment<br>☐ No |
| 6 | Outcome Measures | For all experimental outcomes presented, are details provided of exactly what parameter was measured? | ☒ Yes, for at least one experiment<br>☐ No |
| 7 | Statistical Methods | Is the statistical approach used to analyse each outcome detailed? | ☒ Yes, for at least one analysis<br>☐ No |
| | | Is there a description of any methods used to assess whether data met statistical assumptions? | ☒ Yes, for at least one analysis<br>☐ No<br>☐ Not applicable |
| 8 | Experimental Animals | Are all species of animal used specified? | ☒ Yes, for at least one experiment<br>☐ No |
| | | Is the sex of the animals specified? | ☒ Yes, for at least one experiment<br>☐ No<br>☐ Not applicable to species |
| | | Is at least one of age, weight or developmental stage of the animals specified? | ☒ Yes, for at least one experiment<br>☐ No |
| 9 | Experimental Procedures | Are both the timing and frequency with which procedures took place specified? | ☒ Yes, for at least one experiment<br>☐ No |
| | | Are details of acclimatisation periods to experimental locations provided? | ☒ Yes, for at least one experiment<br>☐ No |
| 10 | Results | Are descriptive statistics for each experimental group provided, with a measure of variability (e.g. mean and SD, or median and range)? | ☒ Yes, for at least one experiment<br>☐ No<br>☐ Not applicable to the type of data collected |
| | | Is the effect size and confidence interval provided? | ☒ Yes, for at least one experiment<br>☐ No<br>☐ Not applicable to the type of analysis used |
